# A Novel Family of *Acinetobacter* Mega-Plasmids Are Disseminating Multi-Drug Resistance Across the Globe While Acquiring Location-Specific Accessory Genes

**DOI:** 10.3389/fmicb.2020.605952

**Published:** 2020-12-02

**Authors:** Timothy M. Ghaly, Ian T. Paulsen, Ammara Sajjad, Sasha G. Tetu, Michael R. Gillings

**Affiliations:** ^1^Department of Biological Sciences, Macquarie University, Sydney, NSW, Australia; ^2^Department of Molecular Sciences, Macquarie University, Sydney, NSW, Australia; ^3^ARC Centre of Excellence in Synthetic Biology, Macquarie University, Sydney, NSW, Australia

**Keywords:** multi-drug resistance, pathogens, mobile genetic elements, nosocomial infections, plasmid pangenome

## Abstract

*Acinetobacter* species are emerging as major nosocomial pathogens, aided by their ability to acquire resistance to all classes of antibiotics. A key factor leading to their multi-drug resistance phenotypes is the acquisition of a wide variety of mobile genetic elements, particularly large conjugative plasmids. Here, we characterize a family of 21 multi-drug resistance mega-plasmids in 11 different *Acinetobacter* species isolated from various locations across the globe. The plasmid family exhibits a highly dynamic and diverse accessory genome, including 221 antibiotic resistance genes (ARGs) that confer resistance to 13 classes of antibiotics. We show that plasmids isolated within the same geographic region are often evolutionarily divergent members of this family based on their core-genome, yet they exhibit a more similar accessory genome. Individual plasmids, therefore, can disseminate to different locations around the globe, where they then appear to acquire diverse sets of accessory genes from their local surroundings. Further, we show that plasmids from several geographic regions were enriched with location-specific functional traits. Together, our findings show that these mega-plasmids can transmit across species boundaries, have the capacity for global dissemination, can accumulate a diverse suite of location-specific accessory genes, and can confer multi-drug resistance phenotypes of significant concern for human health. We therefore highlight this previously undescribed plasmid family as a serious threat to healthcare systems worldwide. These findings also add to the growing concern that mega-plasmids are key disseminators of antibiotic resistance and require global surveillance.

## Introduction

*Acinetobacter* is a diverse genus of bacteria that commonly occur in soil and water, and in association with animals and plants (Fondi et al., 2010). Several species are emerging as major nosocomial pathogens (Almasaudi, 2018). In particular, *Acinetobacter baumannii*, *Acinetobacter nosocomialis*, *Acinetobacter pitti*, *Acinetobacter ursingii*, *Acinetobacter haemolyticus*, and *Acinetobacter calcoaceticus* are now a serious threat to human health (Antunes et al., 2014; Chusri et al., 2014; Weber et al., 2016; Castro-Jaimes et al., 2020; Rivera-Izquierdo et al., 2020). Clinical isolates are often characterized by their multi-drug resistance, with some isolates being resistant to all classes of antibiotics (Göttig et al., 2014). One of the key features leading to their multi-drug resistance is the acquisition of a wide variety of mobile DNA elements (Fournier et al., 2006; Hamidian et al., 2014, 2016; Lean and Yeo, 2017). In particular, *Acinetobacter* strains are known to harbor a strikingly diverse pool of plasmids (Fournier et al., 2006; Fondi et al., 2010; Merino et al., 2010; Lean and Yeo, 2017; Salto et al., 2018).

The evolutionary and ecological dynamics of *Acinetobacter* plasmids are particularly interesting as their host species can colonize a wide variety of environments and display diverse metabolic activities, even among closely related strains (Hawkey and Munday, 2004; Bach and Gutnick, 2006; Albarracín et al., 2012; Garcia-Garcera et al., 2017). Such variety in niche adaptation is largely driven by the open pangenome of their plasmids (Fondi et al., 2010). Plasmids, in general, can rapidly diversify through a series of recombinatorial and transpositional events that generate novel mosaic elements (Stokes and Gillings, 2011). These mosaic elements are characterized by a nested structure of diverse mobile elements, including transposons and “clinical” forms of integrons (Ghaly et al., 2017; Gillings, 2017), each of which are prolific at acquiring diverse antibiotic resistance genes (ARGs; Babakhani and Oloomi, 2018; Ghaly et al., 2020). Humans, largely through the use of various selective agents, such as heavy metals, disinfectants, and antibiotics, are increasing the frequency of such occurrences (Gillings and Stokes, 2012; Gillings et al., 2018). The same selective agents also upregulate the horizontal transmission mechanisms of these mosaic elements, leading to the wide-spread dissemination of their genetic cargo (Beaber et al., 2004; Schreiber et al., 2013; Ghaly and Gillings, 2018). In clinical settings, this has led to the emergence of multi-drug resistant pathogens that are well adapted to a nosocomial lifestyle.

An extensive range of resistance genes in *Acinetobacter* are plasmid-borne (Cerezales et al., 2020). In particular, large plasmids of more than 100 kb, referred to as mega-plasmids, are known to carry arrays of multiple resistance genes (Hamidian et al., 2016; Nigro and Hall, 2017). Mega-plasmids, in general, are characterized by their mosaic structure and carry genetic regions that originate from various ancestral sources (Pesesky et al., 2019). Recently, a family of mega-plasmids has been identified as a major disseminator of multi-drug resistance among *Pseudomonas* species (Cazares et al., 2020). These mega-plasmids carried large arrays of complex and dynamic resistance regions, suggesting that they play a key role in the spread of resistance.

Global surveillance of such mega-plasmids using a whole-genome sequencing approach has been highlighted as a top priority for public health management (Cazares et al., 2020). However, fully sequencing these mega-plasmids with short-read data remains difficult as they contain long repeat regions (Arredondo-Alonso et al., 2017). Long-read sequencing, however, allows these mega-plasmids to be fully resolved (Botelho et al., 2019). The accuracy of assemblies can be improved by combining short-read and long-read sequencing approaches.

Here, we use a combination of PacBio long-read and Illumina short-read sequencing to fully assemble four multi-resistance mega-plasmids resident in both clinical and environmental *Acinetobacter* strains. We collected an additional 17 publicly available complete plasmid sequences within this family, isolated from various locations across the world. This previously undescribed mega-plasmid family, found in 11 different *Acinetobacter* species so far, shares a tight core-genome, yet varies extensively in its accessory genome. By characterizing its pangenome, we show that this plasmid family can rapidly disperse around the globe, where it subsequently accumulates a diverse suite of niche-adaptive accessory genes.

## Materials and Methods

### Sampling, DNA Extraction, and Sequencing

The novel plasmids described in this study were recovered from *Acinetobacter* species. Three were isolated from *Acinetobacter* strains inhabiting the digestive tracts of wild-caught prawns, harvested from the Australian east coast (pR4WN-1BD1, pR4WN-12CE1, and pR4WN-E10B), and one from an ICU patient at Westmead Hospital, Sydney, Australia (pWM98B). The method by which each *Acinetobacter* host was isolated from the wild-caught prawns (Gillings et al., 2009; Sajjad et al., 2011) and the Westmead Hospital patient (Valenzuela et al., 2007) have been previously described. These bacterial isolates were of interest because they showed sequence similarity in the genomic landscape flanking a previously described class 1 integron (Gillings et al., 2009). To investigate this further, DNA was extracted from pure cultures using a standard phenol:chloroform protocol (Sambrook and Russell, 2006). Whole genome *de novo* sequencing was performed using both long-read and 100 bp paired-end sequencing on the PacBio® *RS* and the Illumina® HiSeq 2500 systems, respectively. Sequencing was carried out at the Macrogen Sequencing Facility (Seoul, South Korea).

### Plasmid Assembly and Polishing

PacBio long-reads were assembled with Flye v2.7.1 (Kolmogorov et al., 2019) with three iterations of Flye’s in-built polisher (parameters: –plasmids -i 3). The Flye assemblies were then further polished with the HiSeq paired-end reads using Pilon v1.23 (Walker et al., 2014). Polishing with Pilon first involved mapping all paired-end reads to the PacBio assemblies using BWA v0.7.12-r1039 (Li and Durbin, 2009). The BWA mapping was then used by Pilon to polish the assemblies. Several rounds of polishing (3–4 iterations) were implemented until no further changes were made to the previous round. Polished contigs were then identified to be circular using Circlator v1.5.5 (Hunt et al., 2015), which outputs a linearized version of each circular contig (parameters: all -- assembler canu). Circularized contigs were then re-polished with the paired-end reads using Pilon. All assemblies required a further two rounds of Pilon polishing until no changes were made to the previous round.

### Taxonomic Labeling and Collecting Related Plasmids

Contigs representing the complete chromosome from each assembly were assigned a taxonomy by best BLASTn match using BLAST v2.7.1 (Madden, 2013) against all complete bacterial genomes in RefSeq (downloaded: June 25, 2020). Taxonomic identification was then confirmed with Kraken v1.0 (Wood and Salzberg, 2014; parameters: -- threads 24).

From each of the four samples, a complete sequence was also assembled for the plasmid carrying the integron of interest. Sequence coverage statistics for each of these were generating by mapping sequence reads to each plasmid assembly using Minimap2 v2.17-r941 (Li, 2018) with the parameters (-ax map-pb) and (-ax sr) for PacBio and Illumina mapping, respectively. Sequence coverage details were then extracted from each read mapping using BEDTools v2.27.1 (Quinlan and Hall, 2010; parameters: genomeCoveragBed).

To identify all related plasmids that had previously been sequenced and submitted to NCBI, each plasmid was used to query the complete nr/nt database as of June 26, 2020^1^ using BLASTn. All plasmids that were considered related had a BLAST query cover greater than 64% and a percent identity greater than 98.5%. This resulted in an additional 17 plasmids for inclusion in the detailed analyses below (GenBank accessions: CP010351, CP029396, CP032285, CP033130, CP033531, CP033569, CP038010, CP042365, CP042557, CP043053, CP043309, CP048828, KX426227, MH220285 – MH220287, and MK134375). Plasmid alignment visualizations were generated using Chromatiblock v0.4.2 (Sullivan and van Bakel, 2019) and AliTV v1.0.6 (Ankenbrand et al., 2017).

To determine to which Rep family the plasmids belonged, we identified replication initiation proteins by annotation with InterProScan v5.44-79.0 (Jones et al., 2014; parameters: -goterms) and by BLASTP alignments with representative Rep proteins from every known *Acinetobacter* Rep family (available as Supplementary Figure S2 from Salgado-Camargo et al., 2020).

### Plasmid Pan‐ and Core-Genome Analyses

Gene and protein sequences for all 21 plasmids were predicted using Prokka v1.13 (parameters: -- kingdom Bacteria). Plasmid pan‐ and core-genome analyses were carried out using Perl scripts available in the GET_HOMOLOGUES v20092018 software package (Contreras-Moreira and Vinuesa, 2013; Vinuesa and Contreras-Moreira, 2015). For the plasmid pan-genome, this involved generating two pangenome matrices based on the COGtriangles (Kristensen et al., 2010) and OMCL (Li et al., 2003) clustering algorithms using the get_homologues.pl script. The parameters (-X -c -n 8 -t 0 -A -G) and (-X -c -n 8 -t 0 -A -G -M) were implemented, respectively. Based on the intersection of the two sets of clustered sequences, a consensus pangenome matrix was obtained using the compare_clusters.pl script (parameters: -m -T). The identified plasmid core-genome was based on the intersection of the BDBH, COGtriangles, and OMCL clustering algorithms using the get_homologues.pl script with parameters (-t 21 -e), (-G t0), and (-M -t 0), respectively.

The plasmid pangenome was divided into four occupancy classes. The core-genome was made up of the “soft core” (Kaas et al., 2012), which consisted of the genes found in >95% of plasmids, and the “core,” which contained genes found in all plasmids. The accessory genome was made up of the “cloud” (Wolf et al., 2012), which contained the rare genes found in only one to two plasmids, and the “shell” (Wolf et al., 2012), which comprised the remaining, moderately conserved genes.

The primary Perl script, get_homologues.pl with the above parameters, also estimated the minimum core-genome size for this family of plasmids. The estimate was based on 10 random samples of the 21 plasmids with a fitted curve following the function proposed by Tettelin et al. (2005).

To generate a maximum-likelihood tree that clustered plasmids based on the presence and absence of all accessory genes in the pangenome, the pangenome matrix, reduced to a binary format, was used. The tree was generated using IQ-TREE v1.6.12 (Nguyen et al., 2015; Hoang et al., 2018) with 1,000 bootstrap replicates (parameters: -alrt 1000 -bb 1000). The resulting tree was annotated with FigTree v.1.4.3 (Rambaut, 2017) with midpoint root.

### Phylogenetic Analysis

The evolutionary relationship between plasmids was inferred from the plasmid core-genome. Appropriate phylogenetic loci were identified using the GET_PHYLOMARKERS software package (Vinuesa et al., 2018) with default parameters. The GET_PHYLOMARKERS uses the single-copy plasmid core-genome clusters identified by GET_HOMOLOGUES and removes recombinant sequences as well as loci that produce outlier phylogenies. This resulted in eight high-quality phylogenetic markers. These included genes that encode a TraX conjugative transfer protein, an integrase, three membrane-associated proteins, and three hypothetical proteins of unknown functions. The nucleotide sequences were concatenated and aligned using MAFFT v7.271 (Katoh and Standley, 2013; paramters: -localpair -maxiterate 1000). The best-fit substitution model to suit the alignment was determined using ModelFinder (Kalyaanamoorthy et al., 2017), and a maximum-likelihood tree was generated using IQ-TREE v1.6.12 (Nguyen et al., 2015; Hoang et al., 2018) with 1,000 bootstrap replicates (parameters: -m MFP -alrt 1000 -bb 1000). The resulting tree was annotated with FigTree v.1.4.3 (Rambaut, 2017) with midpoint root.

To test whether plasmids have transmitted horizontally across species boundaries, a co-phylogeny was generated to compare host cell and plasmid phylogenies. We used a whole-genome-based phylogeny of the host chromosomes using the GET_PHYLOMARKERS software package (Vinuesa et al., 2018) as described above. Five of the plasmids retrieved from GenBank did not have a corresponding host chromosome sequence publicly available and were thus excluded from the co-phylogenetic analysis. A co-phylogeny comparing the plasmids and their respective host strains was produced using the R package phytools v0.7-47 (Revell, 2012), which rotates the nodes of both trees to optimize congruence between the tips.

Tree topologies were quantitatively compared using a normalized PH85 tree topological distance (nPH85; Geoghegan et al., 2017). This was used to compare the two plasmid trees based on their core and accessory genomes, respectively, as well as to compare the plasmid and host co-phylogenies. The nPH85 distance quantifies the number of bipartitions that are not shared between the two trees. This is done by randomizing the tip labels for one of the trees, and calculating the PH85 for each replicate to generate a null distribution of the tree topology distance. The nPH85 metric ranges from 0, for identical trees, to 1 for trees that have no clades in common. We calculated nPH85 using the R package NELSI v0.2 (Ho et al., 2015) with 1,000 randomizations, which is shown to be robust even for very large trees (Geoghegan et al., 2017).

### Gene Ontology Enrichment Analysis

A gene ontology (GO) enrichment analysis was done to investigate what functional traits were prevalent among the plasmids from each geographic region. To do this, all proteins encoded by the plasmid pangenome were annotated based on the terms from the GO database (Ashburner et al., 2000; The Gene Ontology Consortium, 2019) using InterProScan v5.44-79.0 (Jones et al., 2014; parameters: -goterms). Proteins from plasmids in each geographic region were tested for GO terms that were significantly enriched relative to the complete plasmid pangenome using WEGO v2.0 (Ye et al., 2018).

### Screening for Antibiotic, Biocide and Metal Resistance Genes, and IS Elements

Antibiotic resistance genes were detected using ABRicate v0.8 (Seemann, 2018) using default parameters. To determine which ARGs were part of integron gene cassettes, IntegronFinder v1.5.1 (Cury et al., 2016) was used (parameters: -local_max -func_annot). We used CARD (Alcock et al., 2020) to confirm the class of antibiotic to which each gene conferred resistance. To screen for metal and biocide resistance genes, the complete manually-curated BactMet2 database (Pal et al., 2014) was used. All proteins were aligned against the BacMet2 database using DIAMOND v0.8.33.95 (Buchfink et al., 2015) with a cut-off alignment criteria of e-value 1 × 10^−5^, 80% similarity, and 90% subject cover length (parameters: --evalue 0.00005 --id 80 --subject-cover 90 --max-target-seqs 1).

### Sequence Data Availability

The complete sequences of the four novel plasmids described here are available in GenBank as accessions MT742180 – MT742183. The complete chromosome sequences of their respective hosts are also available in GenBank as accessions CP059078 – CP059081.

## Results and Discussion

### Plasmid Family and Host Species

Using a combination of PacBio and Illumina sequencing, we generated complete assemblies of four novel mega-plasmids, all of which were recovered from *Acinetobacter* species. Each plasmid had a PacBio sequence coverage > 300x and an Illumina sequence coverage > 500x, except for pWM98B (46x Illumina coverage). Three of these (pR4WN-1BD1, pR4WN-12 CE1, and pR4WN-E10B) were isolated from the digestive tracts of wild-caught prawns and one (pWM98B) from an ICU patient. Another 17 related *Acinetobacter* plasmids were identified in the GenBank database, isolated from various locations across the world and were included in all subsequent analyses. The 21 mega-plasmids were ranged from 250 to 400 kb in size and were harbored by 11 different *Acinetobacter* species (see Supplementary Table S1 for more detailed plasmid information and taxonomy of host bacteria).

We have identified four of the 21 plasmids (pXBB1-9, pAHTJR1, p34AB, and pOXA23_010062) to be part of the Rep-3 superfamily group, based on their replication initiation (Rep) protein. Intriguingly, no Rep protein could be identified in any of the other plasmids, suggesting that it is not needed for successful replication. Similarly, other *Acinetobacter* plasmids, notably pRAY-type plasmids, have no known Rep protein (Hamidian et al., 2012; Lean and Yeo, 2017). It has been proposed that these *rep*-lacking *Acinetobacter* plasmids may use their host machinery for DNA melting and primase activity during plasmid replication, as is the case in ColE1-type plasmids (Lean and Yeo, 2017). It is possible that these mega-plasmids have acquired this replication strategy and subsequently lost their *rep* gene.

### Plasmid Pan‐ and Core-Genome Analyses

**Figure 1:**
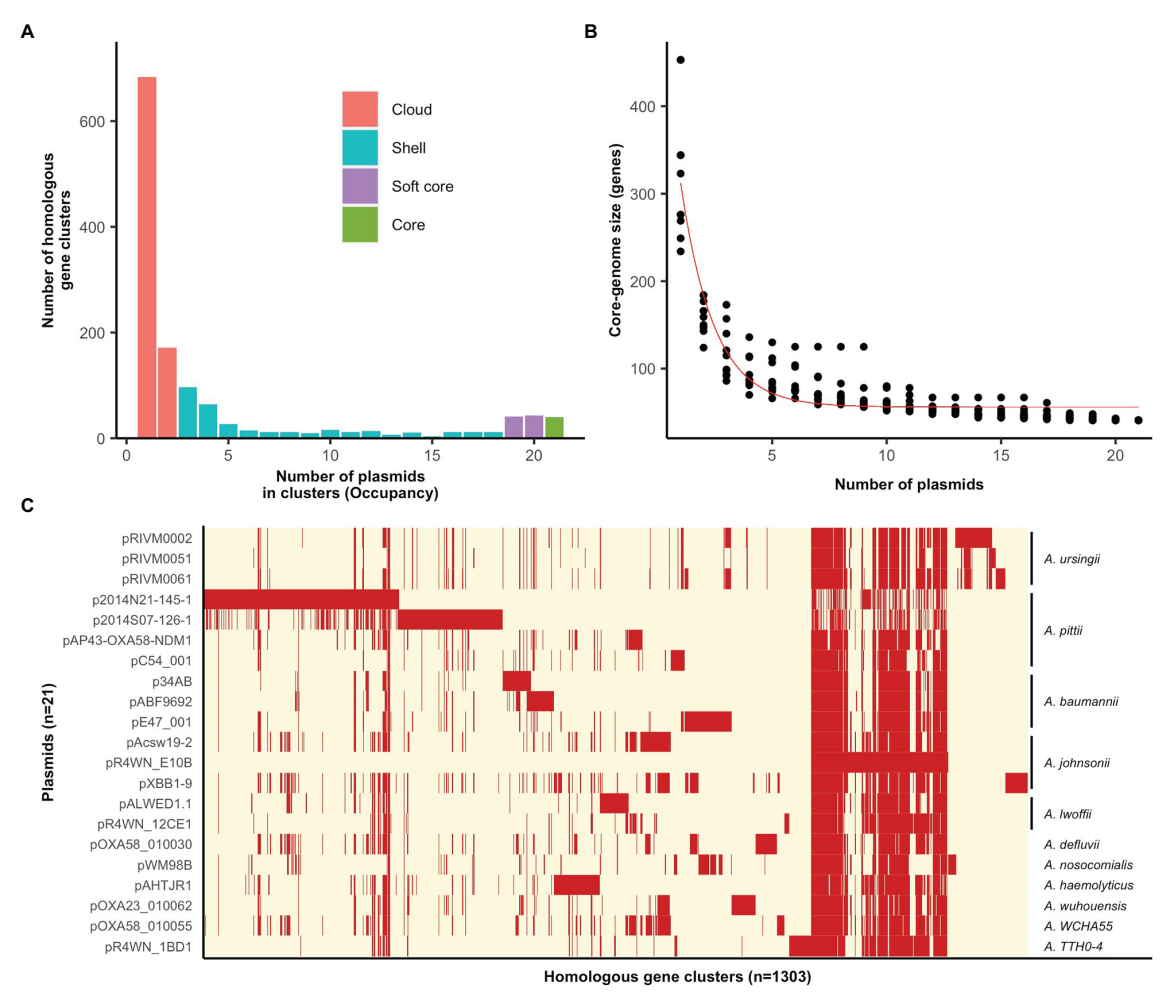
Pan‐ and core-genomic analyses of the mega-plasmid family. **(A)** The plasmid pangenome can be divided into occupancy classes based on the number of plasmids contributing to each homologous gene cluster. The majority of genes belong to the accessory genome, constituting the cloud and shell classes. **(B)** The estimate of the coregenome size (genes) for this plasmid family was generated from 10 random samples of the 21 plasmids. The fitted curve follows the function proposed by Tettelin et al. (2005). **(C)** A pangenome matrix showing the presence (red) and absence (cream) of genes among the plasmids. The *Acinetobacter* species in which each plasmid was resident in are annotated on the right. Plasmids share a tight core-genome, yet, vary extensively in their accessory genomes.

The plasmid pangenome, comprising 1,303 homologous gene clusters, was divided into four occupancy classes (see the section “Methods” for definitions), where the “core” and “soft core” classes make up the core-genome and the “shell” and “cloud” classes make up the accessory genome (Figure 1A). The vast majority of genes carried by this plasmid family are accessory genes (Figures 1A,C), with a minimum set of 40 genes comprising the plasmid core-genome (Figures 1B,C). Individual plasmids carried 95–340 accessory genes each (Supplementary Table S1). It is thus evident that these mega-plasmids have the capacity to accumulate large and diverse sets of accessory genes that drives their diversification.

**Figure 2:**
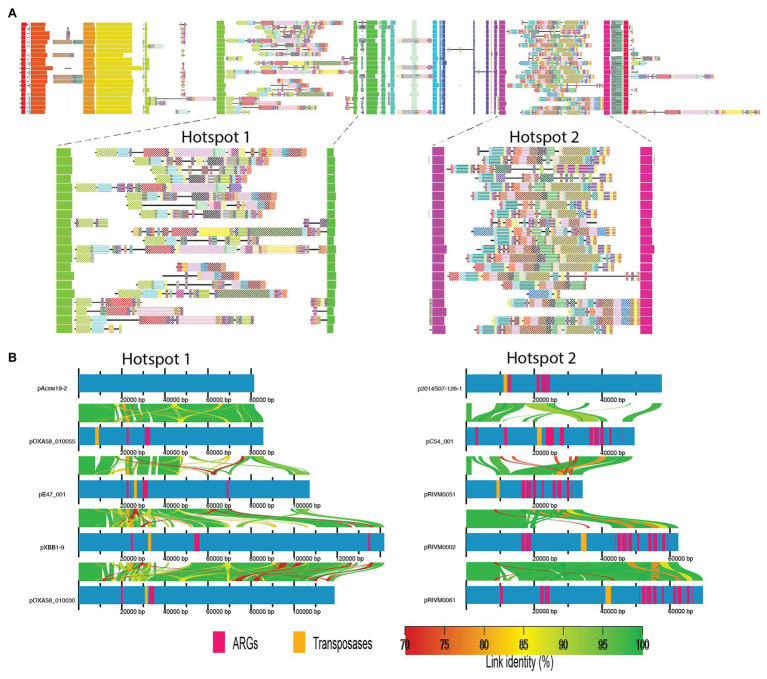
Alignment of the mega-plasmid family. **(A)** A global alignment view of the complete plasmid sequences in this family. Each row represents a single plasmid. Core genomic blocks are visualized as vertically aligned solid rectangles that are colored according to their position in the genome. Non-core blocks are visualized as patterned rectangles. Sequences unique to a single plasmid are depicted as solid black lines. Note that, there are two “hotspots” of considerable genomic variability, labeled as “Hotspot 1” and “Hotspot2.” **(B)** A local alignment view of each hotspot region from five example plasmids. Homologous regions are shown by links that are colored according to their percent nucleotide identity. Locations of antibiotic resistance genes (ARGs) and transposases are annotated on each plasmid. Interestingly, Hotspot 2, on average, constitutes less than 20% of each plasmid, yet contains greater than 65% of all ARGs among these plasmids. Note that, these hotspots have undergone a significant amount of insertions, deletions, and rearrangements. To view the alignments of all 21 plasmids for hotspots 1 and 2, see Supplementary Figures S1, S2, respectively.

The plasmids consist of a number of core conserved genomic blocks separated by highly variable regions (Figure 2A). In particular, there are two “hotspots” of considerable genomic variability, labeled as “Hotspot 1” and “Hotspot 2” (Figures 2A,B). Hotspots 1 and 2 ranged from 7,000–140,000 bp and 15,000–90,000 bp in length, respectively. Alignments of the two hotspots showed a significant number of insertions, deletions, and large-scale rearrangements (Figure 2B; Supplementary Figures S1, S2). We speculate that these highly dynamic regions represent locations were genomic complexity can arise without interfering with core genes or regulatory networks. Interestingly, ARGs were overrepresented in Hotspot 2. This region, which on average constitutes less than 20% of each plasmid, contains greater than 65% of all ARGs (Supplementary Figures S1, S2).

### Plasmid Phylogeny

**Figure 3:**
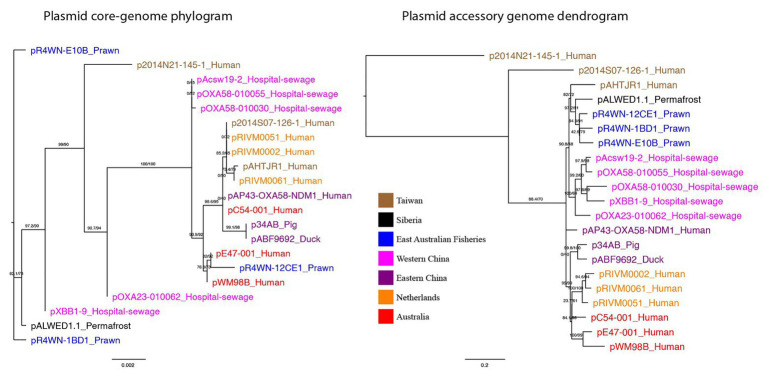
Plasmid core-genome phylogram and accessory genome dendrogram. (left) A maximum-likelihood tree showing the inferred plasmid phylogeny based on the plasmid core-genome and (right) a maximum-likelihood tree based on the presence and absence of all 1,262 accessory genes in the plasmid pangenome. Branch labels signify SH-aLRT support (%)/bootstrap support (%). Plasmids are named in the format of “plasmid-ID”_”Isolation-source” and are colored based on the geographic region from which they were sampled. Together, the two trees show that plasmids from the same geographic region are not necessarily evolutionarily closely related, yet they exhibit a more similar accessory genome.

The evolutionary relationship between plasmids was inferred from the plasmid core-genome using a maximum-likelihood method (Figure 3). In addition, plasmids were clustered based on the presence and absence of all 1,262 accessory genes, also using a maximum-likelihood method (Figure 3). Here, we show that plasmid phylogeny based on core genes does not cluster well according to geographic region. However, based on the presence and absence of all accessory genes, the plasmids do cluster according to their isolation location. Quantitative comparison between the two tree topologies was measured using an nPH85 metric (Geoghegan et al., 2017). The nPH85 metric ranges from 0, for identical trees, to 1, for trees that have no clade bipartitions in common. The accessory and core-genome tree comparison returned an nPH85 value of 0.84, indicating a high-level of incongruence.

Together, these data show that plasmids from the same geographic region are not necessarily evolutionarily closely related, yet they exhibit a more similar accessory genome. This strongly indicates that (a) the plasmids can rapidly disseminate across the globe, given that closely related sister plasmids can be isolated from different parts of the world, and (b) these plasmids preferentially acquire their repertoire of accessory genes from their local environment.

In contrast, the pBT2436-like mega-plasmid family that is driving the dissemination of multi-drug resistance in *Pseudomonas* (Cazares et al., 2020), exhibit similar topologies in their core and accessory genome trees, both of which cluster according to geographic location. This suggests that the *Acinetobacter* mega-plasmids described here have a comparatively greater capacity for global transmission and local acquisition of accessory genes.

**Figure 4:**
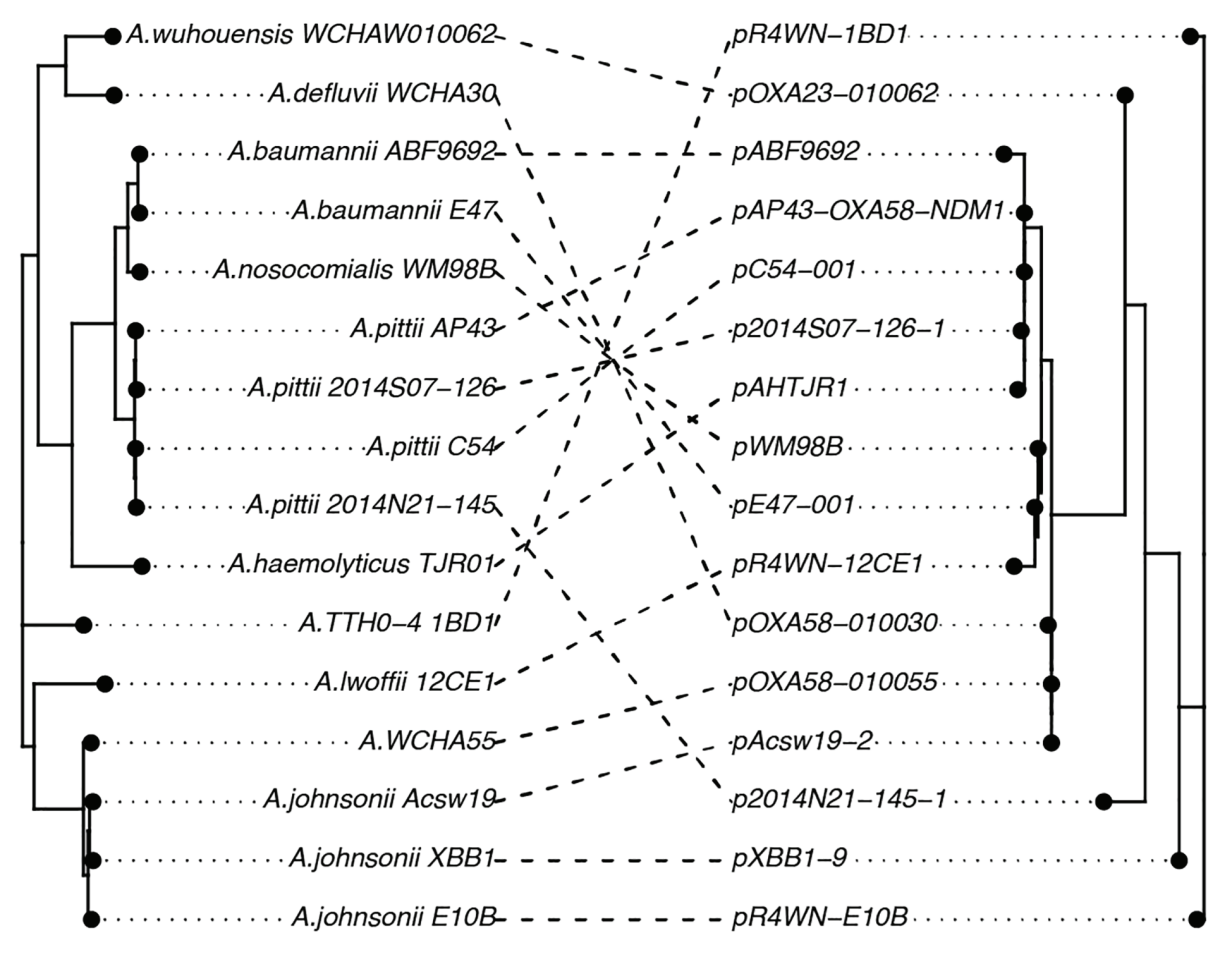
A co-phylogeny linking the host strain (left) and plasmid (right) phylogenies. Connections link host strains with their resident plasmid. Nodes have been rotated to maximize congruency between the two trees. Host phylogeny was inferred from the host core genomes using a maximum-likelihood method. The inferred plasmid phylogeny was based on the plasmid core-genome also using a maximum-likelihood approach. The high level of incongruence between the phylogenies (nPH85 = 1.0) indicates that cross-species transmission is highly prevalent among this family of plasmids.

Further, we show that the topologies of plasmid and host phylogenetic trees are highly incongruent (nPH85 = 1.0; Figure 4). The host and plasmid phylogenies thus share no clade bipartitions in common. Such co-phylogenetic analyses can be used to distinguish between a history of host-plasmid co-divergence (co-phylogenetic congruence) and cross-species transmission (co-phylogenetic incongruence). Our findings provide evidence that cross-species horizontal transmission among *Acinetobacter* species is highly prevalent for this plasmid family.

### Location-Specific Enrichment of Functional Traits

**Figure 5:**
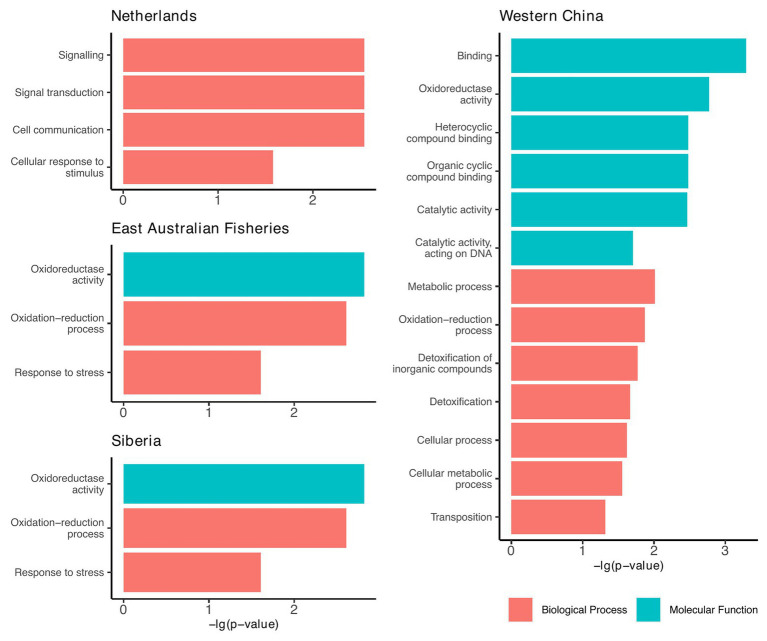
Geographic regions that had plasmids enriched with functional traits relative to the plasmid pangenome. Functions are based on the gene ontology (GO) database and include only those that were significantly enriched compared to the plasmid pangenome (Chi-squared tests, *p* < 0.05).

Since the accessory genomes of the plasmids cluster according to geographic region (Figure 3), we investigated whether there were any functional traits that were prevalent among the plasmids from each region. For this, we annotated proteins based on terms from the GO database. We found that plasmids from four of the seven regions had GO terms that were significantly enriched relative to the plasmid pangenome (Chi-squared tests, *p* < 0.05; Figure 5).

From these results, we can infer the broad-scale selection pressures within each region. For example, plasmids from Western China were largely enriched with genes involved in detoxification, metabolism, and cyclic compound binding (Figure 5). We speculate that these are traits that might be selected in environments of greater pollution. This is particularly significant as the Western China plasmids are polyphyletic (Figure 3), but they share common accessory traits, presumably a result of co-evolution by acquiring accessory genes from the local environment.

### Genetic Cargo of Clinical Relevance

**Figure 6:**
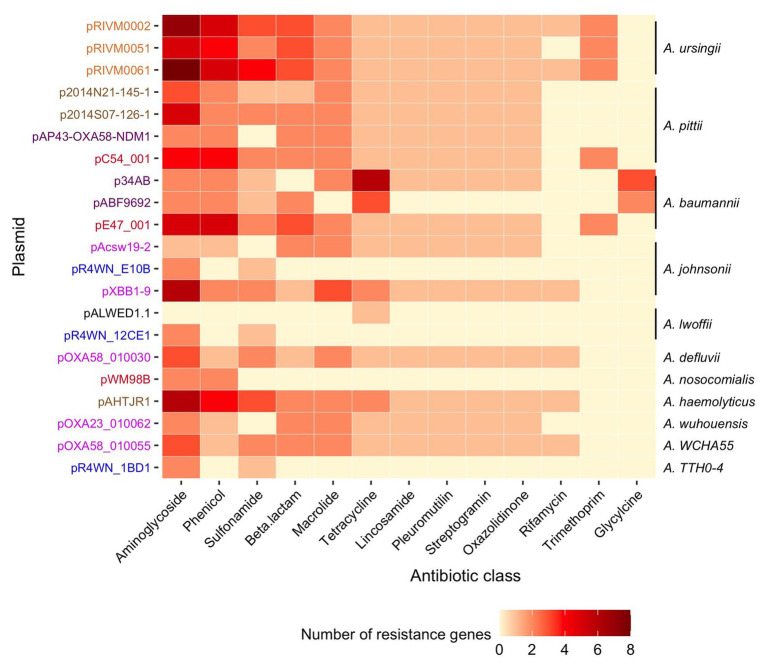
Diversity and abundance of ARGs. The number of resistance genes (color scale bar) is shown for each plasmid (rows). Plasmid names are colored according to the geographic region from which they were sampled, as described in Figure 3. The *Acinetobacter* species in which each plasmid was resident in are annotated on the right. Resistance genes are grouped according to the class of antibiotic that they are known to confer resistance to.

Collectively, the 21 plasmids carried 221 ARGs. All plasmids carried between 3 and 24 ARGs each, except for the plasmid isolated from Siberia (pALWED1.1), which only had one (tetracycline resistance). Together, the 221 genes encoded 35 different proteins known to confer resistance to 13 classes of antibiotics (Figure 6, full list of all ARGs in Supplementary Table S2). Of these, 31% (*n* = 67) were part of gene cassettes associated with class 1 integrons. In total, there were 20 class 1 integrons found in 14 of the plasmids. Consequently, class 1 integrons play a significant role in the accumulation of antibiotic resistance determinants in this plasmid family.

**Figure 7:**
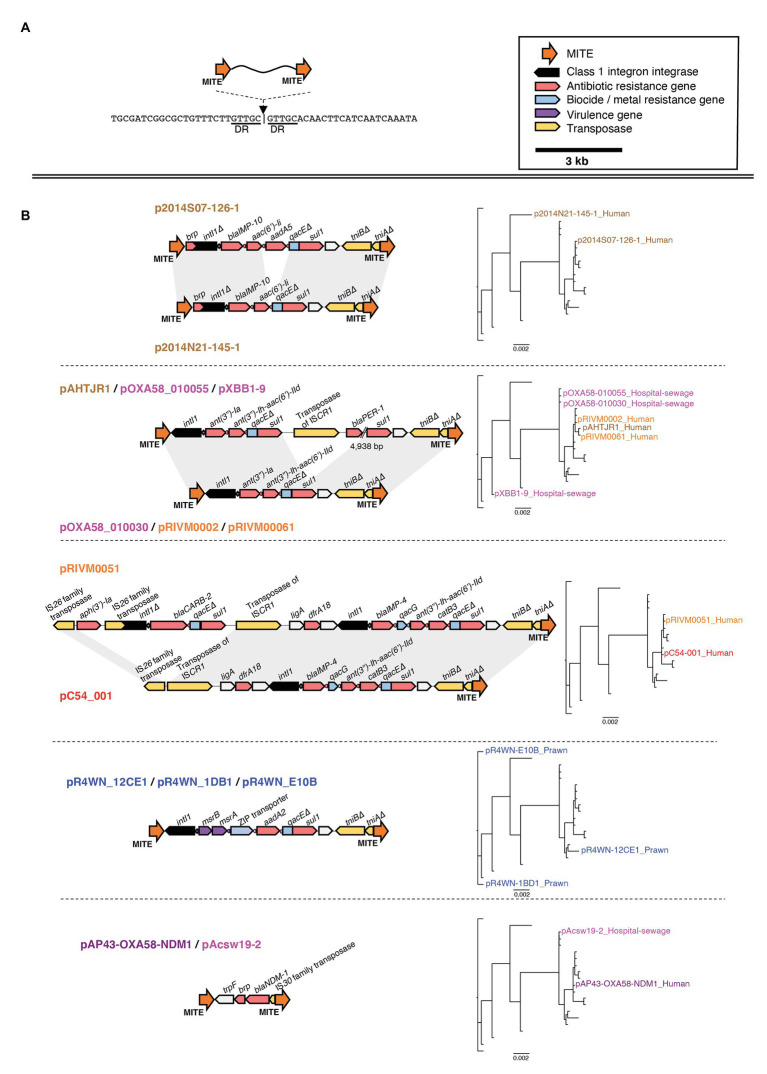
Genetic map of the miniature inverted-repeated transposable element (MITE)-integron complex present among 15 of the 21 plasmids. The insertion site of the MITEs is shown in **(A)**. The structure of each MITE variant is shown in **(B)** and is grouped according to MITE “type.” The plasmids that carry each MITE variant are listed above/below each genetic map. Plasmid names are colored according to the geographic region from which they were sampled, as described in Figure 3. Directional blocks represent genes, while orange arrows represent MITEs as annotated in the top right corner of the figure. All maps are drawn to scale. To the right of each MITE type is the inferred plasmid core-genome phylogeny, highlighting each plasmid that carries the corresponding MITE type. Note that, the same MITE type often occurred in multiple plasmid lineages and could also be present on plasmids from different geographic regions. This suggests that this MITE-resistance region is highly dynamic and transmissible.

In particular, several of the class 1 integrons were associated with the same flanking miniature inverted-repeat transposable elements (MITEs; Figure 7). MITEs are non-autonomous mobile elements that transpose *via* molecular machinery provided *in trans* (Delihas, 2008). Fifteen of the 21 plasmids carried an identical pair of MITEs, each sharing the same insertion site (Figure 7A). Of these, 13 of the MITE pairs were flanking at least one class 1 integron carrying multiple ARG cassettes. Collectively, the MITEs were associated with 15 different ARGs, two virulence factors, and three biocide/metal resistance genes. There were eight unique MITE variants that we grouped into five different MITE “types” based on their cargo of resistance genes (Figure 7B). Interestingly, the same MITE type often occurred in multiple plasmid lineages and could also be present on plasmids from different geographic regions (Figure 7B). This suggests that the MITE-resistance region is highly dynamic and transmissible. Indeed, the same MITE-integron complex has also been detected within a clinical *A. baumannii* strain isolated from a human urine sample in Portugal (Domingues et al., 2011). The Portuguese MITE structure, however, was inserted into a different genomic context, further suggesting its high potential for transmission among *Acinetobacter* strains.

In addition, the plasmids carried 108 genes conferring resistance to bacterial biocides and heavy metals. Together, these genes confer resistance to six heavy metals (Hg, Cu, Au, Cr, Ni, and Co) and nine classes of biocides (acridines, azines, xanthenes, organosulfates, organomercury compounds, quaternary ammonium compounds, biguanides, diamindines, and paraquats). All of the detected metal/biocide resistance genes are part of efflux systems. For a full list of all biocide and metal resistance genes, see Supplementary Table S3.

## Conclusion

The *Acinetobacter* mega-plasmids characterized here appear to be highly proficient in acquiring niche-adaptive accessory genes. Thus, the significant collection of ARGs observed within many of these is likely a selective response to exposure to one or more antibiotics. This is of serious concern, as global antibiotic usage continues to increase, particularly for antibiotics of “last-resort” (Klein et al., 2018). As an often-overlooked consequence of this, antibiotics are polluting the environment, radiating from areas of human populations and agricultural areas (Kümmerer, 2001; Campagnolo et al., 2002). Indeed, environmental concentrations of antibiotics are now often observed within the range of biological and evolutionary significance (Chow et al., 2021). Such intense and ubiquitous antibiotic selection surrounding dense human populations is likely driving the accumulation of diverse ARGs by these plasmids across the globe. Further, it is likely that each plasmid can accrue different suites of resistance genes, reflecting the specific conditions and selection pressures in their particular local environment. Thus, our findings highlight this plasmid family as a serious threat to successful bacterial infection control and human health, particularly for nosocomial infections. These findings also add to the growing concern that mega-plasmids are key disseminators of antibiotic resistance and require wide-spread surveillance (Cazares et al., 2020).

We have characterized a family of multi-drug resistance *Acinetobacter* mega-plasmids. We present evidence that these plasmids are highly transmissible, have the capacity for dispersing at a global-scale, can accumulate a vast cargo of niche-adaptive accessory genes, and can confer multi-drug resistance phenotypes of significant concern for human health. These findings add to a growing idea in the literature that points to the importance of viewing the antibiotic resistance crisis from a mobile element-centred outlook as opposed to a host cell-centric point of view (Lopatkin et al., 2017; Stevenson et al., 2017; Ghaly and Gillings, 2018; Pinilla-Redondo et al., 2018). Thus, expanding our therapeutic focus to consider ways of interfering with the transmission of mobile DNAs may be provide an additional, fruitful means of combating the spread of antibiotic resistance (Ghaly and Gillings, 2018).

## Data Availability Statement

The datasets presented in this study can be found in online repositories. The names of the repository/repositories and accession number(s) can be found in the article/Supplementary Material.

## Author Contributions

TG contributed to sample processing and collection, contributed to the conception and design of analyses, performed the analyses, wrote the original draft of manuscript, and contributed to the final editing of manuscript. IP contributed to the conception and design of analyses and contributed to the final editing of manuscript. AS contributed to sample processing and collection and contributed to the final editing of manuscript. ST contributed to the conception and design of analyses and contributed to the final editing of manuscript. MG contributed to sample processing and collection, contributed to the conception and design of analyses, performed funding acquisition, and contributed to the final editing of manuscript. All authors contributed to the article and approved the submitted version.

### Conflict of Interest

The authors declare that the research was conducted in the absence of any commercial or financial relationships that could be construed as a potential conflict of interest.

## Footnotes

^1^http://www.ncbi.nlm.nih.gov/BLAST/
