# Supplementary material for: A Novel Family of Acinetobacter Mega-Plasmids Are Disseminating Multi-Drug Resistance Across the Globe While Acquiring Location-Specific Accessory Genes
Source: Front Microbiol. 2020 Dec 2;11:605952. doi: 10.3389/fmicb.2020.605952 (PMC7738440; doi:10.3389/fmicb.2020.605952)
Supplement: Supplementary file 1 [file Data_Sheet_1.docx]

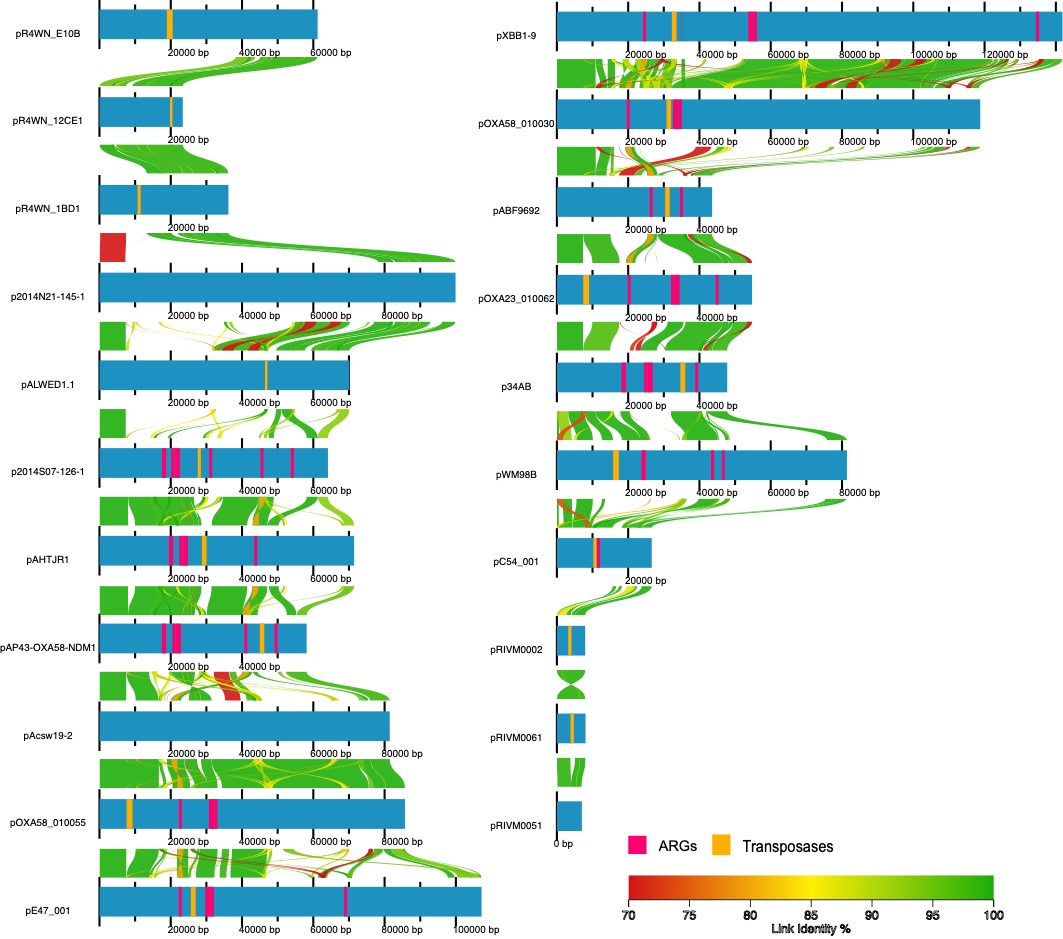


**Fig. S1. A local alignment view of the highly variable ‘Hotspot 1’ region**. Homologous regions are shown by links that are coloured according to their percent identity. Locations of antibiotic resistance genes (ARGs) and transposases are annotated on each plasmid. Note, this hotspot has undergone a significant amount of insertions, deletions and rearrangements.


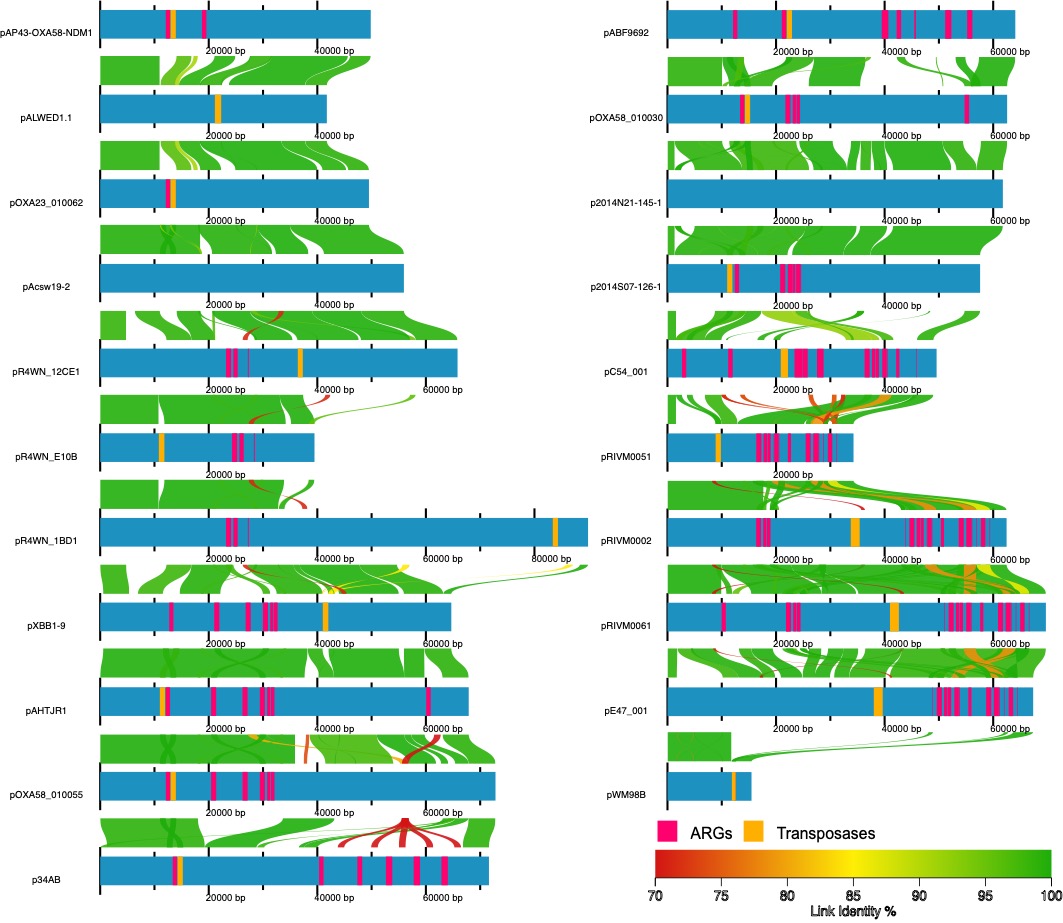


**Fig. S2. A local alignment view of the highly variable ‘Hotspot 2’ region**. Homologous regions are shown by links that are coloured according to their percent identity. Locations of antibiotic resistance genes (ARGs) and transposases are annotated on each plasmid. Interestingly, Hotspot 2, on average, constitutes less than 20% of each plasmid, yet contains greater than 65% of all antibiotic resistance genes among these plasmids. Note, this hotspot has undergone a significant amount of insertions, deletions and rearrangements.

**Table S1. Plasmid details and host taxonomy** (***** Denotes newly sequenced plasmids)

| **Plasmid name** | **GenBank accession** | **Host species** | **Sample source** | **Sample location** | **Plasmid size (bp)** | **Core genes** | **Soft core genes** | **Shell genes** | **Cloud genes** | **Plasmid GC (%)** | **Host GC (%)** |
| --- | --- | --- | --- | --- | --- | --- | --- | --- | --- | --- | --- |
| p34AB | MK134375 | *Acinetobacter baumannii* | Pig | Eastern China | 277864 | 40 | 124 | 73 | 31 | 39.19 | N/A |
| pAP43-OXA58-NDM1 | CP043053 | *Acinetobacter pittii* | Human | Eastern China | 268263 | 40 | 114 | 115 | 10 | 38.63 | 38.87 |
| pABF9692 | CP048828 | *Acinetobacter baumannii* | Duck | Eastern China | 264805 | 40 | 124 | 77 | 35 | 39.16 | 38.99 |
| pAcsw19-2 | CP043309 | *Acinetobacter johnsonii* | Hospital sewage | Western China | 351885 | 40 | 124 | 168 | 14 | 38.79 | N/A |
| pXBB1-9 | CP010351 | *Acinetobacter johnsonii* | Hospital sewage | Western China | 398857 | 40 | 124 | 182 | 56 | 39.99 | 41.43 |
| pOXA58_010055 | CP032285 | *Acinetobacter sp. WCHA55* | Hospital sewage | Western China | 372328 | 40 | 124 | 174 | 20 | 39.28 | 41.42 |
| pOXA58_010030 | CP029396 | *Acinetobacter defluvii* | Hospital sewage | Western China | 355358 | 40 | 123 | 146 | 36 | 39.51 | 37.96 |
| pOXA23_010062 | CP033130 | *Acinetobacter wuhouensis* | Hospital sewage | Western China | 311749 | 40 | 120 | 118 | 38 | 38.38 | 38.09 |
| pRIVM0061 | MH220287 | *Acinetobacter ursingii* | Human | Netherlands | 313407 | 40 | 124 | 127 | 21 | 39.3 | N/A |
| pRIVM0051 | MH220286 | *Acinetobacter ursingii* | Human | Netherlands | 259278 | 40 | 123 | 88 | 8 | 39.28 | N/A |
| pRIVM0002 | MH220285 | *Acinetobacter ursingii* | Human | Netherlands | 317191 | 40 | 123 | 112 | 39 | 39.3 | N/A |
| pALWED1.1 | KX426227 | *Acinetobacter lwoffii* | Permafrost | Siberia | 287631 | 40 | 124 | 86 | 38 | 39.34 | N/A |
| p2014N21-145-1 | CP033569 | *Acinetobacter pittii* | Human | Taiwan | 323995 | 40 | 76 | 102 | 237 | 39.79 | 38.92 |
| pAHTJR1 | CP038010 | *Acinetobacter haemolyticus* | Human | Taiwan | 306131 | 40 | 119 | 112 | 58 | 40.06 | 39.76 |
| p2014S07-126-1 | CP033531 | *Acinetobacter pittii* | Human | Taiwan | 284051 | 40 | 87 | 74 | 199 | 39.44 | 38.85 |
| pC54_001 | CP042365 | *Acinetobacter pittii* | Human | Australia | 256887 | 40 | 117 | 77 | 18 | 38.91 | 38.87 |
| pE47_001 | CP042557 | *Acinetobacter baumannii* | Human | Australia | 327867 | 40 | 124 | 122 | 45 | 40.84 | 39.12 |
| pWM98B***** | MT742183 | *Acinetobacter nosocomialis* | Human | Australia | 255232 | 40 | 119 | 55 | 49 | 41.44 | 38.71 |
| pR4WN_12CE1***** | MT742180 | *Acinetobacter lwoffii* | Prawn | East Australian Fisheries | 270906 | 40 | 123 | 106 | 11 | 39.76 | 43.47 |
| pR4WN_1BD1***** | MT742182 | *Acinetobacter sp. TTH0-4* | Prawn | East Australian Fisheries | 284751 | 40 | 123 | 88 | 39 | 39.17 | 38.69 |
| pR4WN_E10B***** | MT742181 | *Acinetobacter johnsonii* | Prawn | East Australian Fisheries | 259080 | 40 | 120 | 74 | 23 | 38.55 | 41.5 |

**Table S2. The complete list of antibiotic resistance genes detected in each plasmid**

| **Plasmid name** | **Gene name** | **GenBank reference accession** |
| --- | --- | --- |
| p2014N21-145-1 | msr(E)_4 | EU294228 |
| p2014N21-145-1 | mph(E)_3 | EU294228 |
| p2014N21-145-1 | floR_2 | AF118107 |
| p2014N21-145-1 | aph(3')-VIa_1 | X07753 |
| p2014N21-145-1 | sul1_2 | CP002151 |
| p2014N21-145-1 | aac(6')-IIa_1 | M29695 |
| p2014N21-145-1 | blaIMP-10_1 | AB195637 |
| p2014N21-145-1 | ant(3'')-Ia_1 | X02340 |
| p2014S07-126-1 | ant(3'')-Ia_1 | X02340 |
| p2014S07-126-1 | blaIMP-10_1 | AB195637 |
| p2014S07-126-1 | aac(6')-IIa_1 | M29695 |
| p2014S07-126-1 | aadA5_1 | AF137361 |
| p2014S07-126-1 | sul1_2 | CP002151 |
| p2014S07-126-1 | aph(3')-VIa_1 | X07753 |
| p2014S07-126-1 | aac(3)-IId_1 | EU022314 |
| p2014S07-126-1 | blaOXA-58_1 | AY665723 |
| p2014S07-126-1 | sul2_2 | GQ421466 |
| p2014S07-126-1 | msr(E)_4 | EU294228 |
| p2014S07-126-1 | mph(E)_3 | EU294228 |
| p2014S07-126-1 | floR_2 | AF118107 |
| p34AB | tet(X)_1 | GU014535 |
| p34AB | tet(X)_1 | GU014535 |
| p34AB | tet(X)_1 | GU014535 |
| p34AB | tet(Y)_1 | EF495198 |
| p34AB | sul2_2 | GQ421466 |
| p34AB | aph(3')-VIa_1 | X07753 |
| p34AB | aac(3)-IId_1 | EU022314 |
| p34AB | msr(E)_4 | EU294228 |
| p34AB | mph(E)_3 | EU294228 |
| p34AB | floR_2 | AF118107 |
| p34AB | tet(M)_4 | X75073 |
| pABF9692 | tet(M)_4 | X75073 |
| pABF9692 | blaOXA-58_1 | AY665723 |
| pABF9692 | aac(3)-IId_1 | EU022314 |
| pABF9692 | aph(3')-VIa_1 | X07753 |
| pABF9692 | blaNDM-1_1 | FN396876 |
| pABF9692 | floR_2 | AF118107 |
| pABF9692 | sul2_2 | GQ421466 |
| pABF9692 | tet(X)_3 | AB097942 |
| pABF9692 | tet(X)_3 | AB097942 |
| pABF9692 | floR_2 | AF118107 |
| pAHTJR1 | strA_1 | M96392 |
| pAHTJR1 | aph(6)-Id_1 | M28829 |
| pAHTJR1 | tet(Y)_1 | EF495198 |
| pAHTJR1 | catB4_1 | EU935739 |
| pAHTJR1 | aph(3')-Ia_1 | V00359 |
| pAHTJR1 | catB4_1 | EU935739 |
| pAHTJR1 | sul2_2 | GQ421466 |
| pAHTJR1 | ant(3'')-Ia_1 | X02340 |
| pAHTJR1 | ARR-3_4 | FM207631 |
| pAHTJR1 | ant(3'')-Ih-aac(6')-IId_1 | AF453998 |
| pAHTJR1 | sul1_2 | CP002151 |
| pAHTJR1 | blaPER-1_1 | GU944725 |
| pAHTJR1 | sul1_2 | CP002151 |
| pAHTJR1 | aph(3')-VIa_1 | X07753 |
| pAHTJR1 | blaOXA-58_1 | AY665723 |
| pAHTJR1 | msr(E)_4 | EU294228 |
| pAHTJR1 | mph(E)_3 | EU294228 |
| pAHTJR1 | floR_2 | AF118107 |
| pALWED1.1 | tet(H)_3 | Y15510 |
| pAP43-OXA58-NDM1 | blaNDM-1_1 | FN396876 |
| pAP43-OXA58-NDM1 | aph(3')-VIa_1 | X07753 |
| pAP43-OXA58-NDM1 | aac(3)-IId_1 | EU022314 |
| pAP43-OXA58-NDM1 | blaOXA-58_1 | AY665723 |
| pAP43-OXA58-NDM1 | msr(E)_4 | EU294228 |
| pAP43-OXA58-NDM1 | mph(E)_3 | EU294228 |
| pAP43-OXA58-NDM1 | floR_2 | AF118107 |
| pAcsw19-2 | mph(E)_3 | EU294228 |
| pAcsw19-2 | msr(E)_4 | EU294228 |
| pAcsw19-2 | blaOXA-58_1 | AY665723 |
| pAcsw19-2 | blaNDM-1_1 | FN396876 |
| pAcsw19-2 | aph(3')-VIa_1 | X07753 |
| pC54_001 | aac(3)-IId_1 | EU022314 |
| pC54_001 | aph(3')-VIa_1 | X07753 |
| pC54_001 | sul2_2 | GQ421466 |
| pC54_001 | msr(E)_4 | EU294228 |
| pC54_001 | mph(E)_3 | EU294228 |
| pC54_001 | floR_2 | AF118107 |
| pC54_001 | sul1_2 | CP002151 |
| pC54_001 | catB3_1 | AJ009818 |
| pC54_001 | ant(3'')-Ih-aac(6')-IId_1 | AF453998 |
| pC54_001 | dfrA7_1 | AJ419170 |
| pC54_001 | blaIMP-26_1 | GU045307 |
| pC54_001 | ant(3'')-Ia_1 | X02340 |
| pC54_001 | dfrA18_1 | AJ310778 |
| pC54_001 | catB4_1 | EU935739 |
| pC54_001 | blaOXA-58_1 | AY665723 |
| pE47_001 | blaOXA-96_1 | DQ519090 |
| pE47_001 | msr(E)_4 | EU294228 |
| pE47_001 | mph(E)_3 | EU294228 |
| pE47_001 | aac(3)-IId_1 | EU022314 |
| pE47_001 | catB4_1 | EU935739 |
| pE47_001 | sul1_2 | CP002151 |
| pE47_001 | catB3_1 | AJ009818 |
| pE47_001 | ant(3'')-Ih-aac(6')-IId_1 | AF453998 |
| pE47_001 | dfrA7_1 | AJ419170 |
| pE47_001 | blaIMP-4_1 | AF244145 |
| pE47_001 | ant(3'')-Ia_1 | X02340 |
| pE47_001 | dfrA18_1 | AJ310778 |
| pE47_001 | sul1_2 | CP002151 |
| pE47_001 | blaCARB-2_1 | M69058 |
| pE47_001 | ant(3'')-Ia_1 | X02340 |
| pE47_001 | catB4_1 | EU935739 |
| pE47_001 | aph(3')-Ia_1 | V00359 |
| pE47_001 | catB4_1 | EU935739 |
| pOXA23_010062 | aph(3')-VIa_1 | X07753 |
| pOXA23_010062 | blaOXA-23_1 | HQ700358 |
| pOXA23_010062 | blaOXA-58_1 | AY665723 |
| pOXA23_010062 | mph(E)_3 | EU294228 |
| pOXA23_010062 | msr(E)_4 | EU294228 |
| pOXA23_010062 | aac(3)-IId_1 | EU022314 |
| pOXA58_010030 | aph(3')-VIa_1 | X07753 |
| pOXA58_010030 | sul1_2 | CP002151 |
| pOXA58_010030 | ant(3'')-Ih-aac(6')-IId_1 | AF453998 |
| pOXA58_010030 | ARR-3_4 | FM207631 |
| pOXA58_010030 | ant(3'')-Ia_1 | X02340 |
| pOXA58_010030 | sul2_2 | GQ421466 |
| pOXA58_010030 | blaOXA-58_1 | AY665723 |
| pOXA58_010030 | msr(E)_4 | EU294228 |
| pOXA58_010030 | mph(E)_3 | EU294228 |
| pOXA58_010055 | ant(3'')-Ia_1 | X02340 |
| pOXA58_010055 | ARR-3_4 | FM207631 |
| pOXA58_010055 | ant(3'')-Ih-aac(6')-IId_1 | AF453998 |
| pOXA58_010055 | sul1_2 | CP002151 |
| pOXA58_010055 | blaPER-1_1 | GU944725 |
| pOXA58_010055 | sul1_2 | CP002151 |
| pOXA58_010055 | aph(3')-VIa_1 | X07753 |
| pOXA58_010055 | mph(E)_3 | EU294228 |
| pOXA58_010055 | msr(E)_4 | EU294228 |
| pOXA58_010055 | blaOXA-58_1 | AY665723 |
| pR4WN_12CE1 | sul1_2 | CP002151 |
| pR4WN_12CE1 | aadA2_2 | JQ364967 |
| pR4WN_12CE1 | ant(3'')-Ia_1 | X02340 |
| pR4WN_1BD1 | sul1_2 | CP002151 |
| pR4WN_1BD1 | aadA2_2 | JQ364967 |
| pR4WN_1BD1 | ant(3'')-Ia_1 | X02340 |
| pR4WN_E10B | sul1_2 | CP002151 |
| pR4WN_E10B | aadA2_2 | JQ364967 |
| pR4WN_E10B | ant(3'')-Ia_1 | X02340 |
| pWM98B | catB4_1 | EU935739 |
| pWM98B | aph(3')-VIa_1 | X07753 |
| pWM98B | aac(3)-IId_1 | EU022314 |
| pWM98B | floR_2 | AF118107 |
| pRIVM0002 | sul1_2 | CP002151 |
| pRIVM0002 | ant(3'')-Ih-aac(6')-IId_1 | AF453998 |
| pRIVM0002 | ARR-3_4 | FM207631 |
| pRIVM0002 | ant(3'')-Ia_1 | X02340 |
| pRIVM0002 | catB4_1 | EU935739 |
| pRIVM0002 | sul1_2 | CP002151 |
| pRIVM0002 | catB3_1 | AJ009818 |
| pRIVM0002 | ant(3'')-Ih-aac(6')-IId_1 | AF453998 |
| pRIVM0002 | dfrA7_1 | AJ419170 |
| pRIVM0002 | blaIMP-4_1 | AF244145 |
| pRIVM0002 | ant(3'')-Ia_1 | X02340 |
| pRIVM0002 | dfrA18_1 | AJ310778 |
| pRIVM0002 | sul1_2 | CP002151 |
| pRIVM0002 | blaCARB-2_1 | M69058 |
| pRIVM0002 | ant(3'')-Ia_1 | X02340 |
| pRIVM0002 | catB4_1 | EU935739 |
| pRIVM0002 | aph(3')-Ia_1 | V00359 |
| pRIVM0002 | catB4_1 | EU935739 |
| pRIVM0002 | blaOXA-58_1 | AY665723 |
| pRIVM0002 | aac(3)-IId_1 | EU022314 |
| pRIVM0002 | mph(E)_3 | EU294228 |
| pRIVM0002 | msr(E)_4 | EU294228 |
| pRIVM0051 | sul1_2 | CP002151 |
| pRIVM0051 | catB3_1 | AJ009818 |
| pRIVM0051 | ant(3'')-Ih-aac(6')-IId_1 | AF453998 |
| pRIVM0051 | dfrA7_1 | AJ419170 |
| pRIVM0051 | blaIMP-4_1 | AF244145 |
| pRIVM0051 | ant(3'')-Ia_1 | X02340 |
| pRIVM0051 | dfrA18_1 | AJ310778 |
| pRIVM0051 | sul1_2 | CP002151 |
| pRIVM0051 | blaCARB-2_1 | M69058 |
| pRIVM0051 | ant(3'')-Ia_1 | X02340 |
| pRIVM0051 | catB4_1 | EU935739 |
| pRIVM0051 | aph(3')-Ia_1 | V00359 |
| pRIVM0051 | catB4_1 | EU935739 |
| pRIVM0051 | blaOXA-58_1 | AY665723 |
| pRIVM0051 | aac(3)-IId_1 | EU022314 |
| pRIVM0051 | mph(E)_3 | EU294228 |
| pRIVM0051 | msr(E)_4 | EU294228 |
| pRIVM0061 | aph(3')-VIa_1 | X07753 |
| pRIVM0061 | sul1_2 | CP002151 |
| pRIVM0061 | sul1_1 | AY224185 |
| pRIVM0061 | ant(3'')-Ih-aac(6')-IId_1 | AF453998 |
| pRIVM0061 | ARR-3_4 | FM207631 |
| pRIVM0061 | ant(3'')-Ia_1 | X02340 |
| pRIVM0061 | catB4_1 | EU935739 |
| pRIVM0061 | sul1_2 | CP002151 |
| pRIVM0061 | catB3_1 | AJ009818 |
| pRIVM0061 | ant(3'')-Ih-aac(6')-IId_1 | AF453998 |
| pRIVM0061 | dfrA7_1 | AJ419170 |
| pRIVM0061 | blaIMP-4_1 | AF244145 |
| pRIVM0061 | ant(3'')-Ia_1 | X02340 |
| pRIVM0061 | dfrA18_1 | AJ310778 |
| pRIVM0061 | sul1_2 | CP002151 |
| pRIVM0061 | blaCARB-2_1 | M69058 |
| pRIVM0061 | ant(3'')-Ia_1 | X02340 |
| pRIVM0061 | catB4_1 | EU935739 |
| pRIVM0061 | aph(3')-Ia_1 | V00359 |
| pRIVM0061 | catB4_1 | EU935739 |
| pRIVM0061 | aac(3)-IId_1 | EU022314 |
| pRIVM0061 | blaOXA-58_1 | AY665723 |
| pRIVM0061 | mph(E)_3 | EU294228 |
| pRIVM0061 | msr(E)_4 | EU294228 |
| pXBB1-9 | strA_1 | M96392 |
| pXBB1-9 | aph(6)-Id_1 | M28829 |
| pXBB1-9 | tet(Y)_1 | EF495198 |
| pXBB1-9 | catB4_1 | EU935739 |
| pXBB1-9 | ant(3'')-Ia_1 | X02340 |
| pXBB1-9 | ARR-3_4 | FM207631 |
| pXBB1-9 | ant(3'')-Ih-aac(6')-IId_1 | AF453998 |
| pXBB1-9 | sul1_2 | CP002151 |
| pXBB1-9 | blaPER-1_1 | GU944725 |
| pXBB1-9 | sul1_2 | CP002151 |
| pXBB1-9 | aph(3')-VIa_1 | X07753 |
| pXBB1-9 | aac(3)-IId_1 | EU022314 |
| pXBB1-9 | mph(E)_3 | EU294228 |
| pXBB1-9 | msr(E)_4 | EU294228 |
| pXBB1-9 | mph(E)_3 | EU294228 |
| pXBB1-9 | blaOXA-58_1 | AY665723 |

**Table S3. The complete list of metal and biocide resistance genes detected in each plasmid**

| **Plasmid name** | **Gene name** | **GenBank Reference accession** | **Compound(s) to which gene confers resistance** | **Reference NCBI annotation** |
| --- | --- | --- | --- | --- |
| p2014N21-145-1 | adeK | WP_033917515.1 | Acridine Orange [class: Acridine]; Safranin O [class: Azin]; Pyronin Y [class: Xanthene]; Sodium Dodecyl Sulfate (SDS) [class: Organo-sulfate] | MULTISPECIES: adeC/adeK/oprM family multidrug efflux complex outer membrane factor |
| p2014N21-145-1 | merE | WP_047731872.1 | Mercury (Hg) | MULTISPECIES: mercury resistance protein |
| p2014N21-145-1 | merD | WP_033917457.1 | Mercury (Hg) | MULTISPECIES: mercuric resistance transcriptional repressor protein MerD |
| p2014N21-145-1 | merB1 | WP_033917456.1 | Mercury (Hg); Phenylmercury Acetate [class: Organo-mercury] | MULTISPECIES: alkylmercury lyase |
| p2014N21-145-1 | merA | WP_033917455.1 | Mercury (Hg); Phenylmercury Acetate [class: Organo-mercury] | MULTISPECIES: mercury(II) reductase |
| p2014N21-145-1 | merC | WP_035884597.1 | Mercury (Hg) | MULTISPECIES: hypothetical protein |
| p2014N21-145-1 | merR | WP_001166626.1 | Mercury (Hg) | MULTISPECIES: Hg(II)-responsive transcriptional regulator |
| p2014N21-145-1 | merT | WP_005028429.1 | Mercury (Hg) | MULTISPECIES: mercuric ion transporter MerT |
| p2014N21-145-1 | merP | WP_005028432.1 | Mercury (Hg) | MULTISPECIES: mercuric transport protein periplasmic component |
| p2014N21-145-1 | merA | WP_033917451.1 | Mercury (Hg); Phenylmercury Acetate [class: Organo-mercury] | MULTISPECIES: mercury(II) reductase |
| p2014N21-145-1 | merB | ASA06980.1 | Mercury (Hg); Phenylmercury Acetate [class: Organo-mercury]; Methylmercury Acetate [class: Organo-mercury] | alkylmercury lyase |
| p2014N21-145-1 | qacEdelta1 | Q7BQY4 | Benzylkonium Chloride (BAC) [class: Quaternary Ammonium Compounds (QACs)]; Ethidium Bromide [class: Phenanthridine]; Acriflavine [class: Acridine]; Chlorhexidine [class: Biguanides]; Pyronin Y [class: Xanthene]; Rhodamine 6G [class: Xanthene]; Methyl Viologen [class: Paraquat]; Tetraphenylphosphonium (TPP) [class: Quaternary Ammonium Compounds (QACs)]; 4;6-diamidino-2-phenylindole (DAPI) [class: Diamindine]; Acridine Orange [class: Acridine]; Sodium Dodecyl Sulfate (SDS) [class: Organo-sulfate]; Sodium Deoxycholate (SDC) [class: Acid]; Crystal Violet [class: Triarylmethane]; Cetrimide (CTM) [class: Quaternary Ammonium Compounds (QACs)]; Cetylpyridinium Chloride (CPC) [class: Quaternary Ammonium Compounds (QACs)]; Dequalinium [class: Quaternary Ammonium Compounds (QACs)] | Quaternary ammonium compound-resistance protein (QacEdelta1) (QacEdelta1multidrug exporter) (Disinfectant resistance protein) (QacEdelta) (Disinfectant resistance protein qacEdelta1) |
| pAcsw19-2 | merR | WP_001166626.1 | Mercury (Hg) | MULTISPECIES: Hg(II)-responsive transcriptional regulator |
| pAcsw19-2 | merC | WP_035884597.1 | Mercury (Hg) | MULTISPECIES: hypothetical protein |
| pAcsw19-2 | merA | WP_033917455.1 | Mercury (Hg); Phenylmercury Acetate [class: Organo-mercury] | MULTISPECIES: mercury(II) reductase |
| pAcsw19-2 | merB1 | WP_033917456.1 | Mercury (Hg); Phenylmercury Acetate [class: Organo-mercury] | MULTISPECIES: alkylmercury lyase |
| pAcsw19-2 | merD | WP_033917457.1 | Mercury (Hg) | MULTISPECIES: mercuric resistance transcriptional repressor protein MerD |
| pAcsw19-2 | adeK | WP_033917515.1 | Acridine Orange [class: Acridine]; Safranin O [class: Azin]; Pyronin Y [class: Xanthene]; Sodium Dodecyl Sulfate (SDS) [class: Organo-sulfate] | MULTISPECIES: adeC/adeK/oprM family multidrug efflux complex outer membrane factor |
| pAcsw19-2 | adeB | WP_068930996.1 | Pyronin Y [class: Xanthene]; Sodium Dodecyl Sulfate (SDS) [class: Organo-sulfate]; Acriflavine [class: Acridine]; Benzylkonium Chloride (BAC) [class: Quaternary Ammonium Compounds (QACs)]; Chlorhexidine [class: Biguanides]; 4;6-diamidino-2-phenylindole (DAPI) [class: Diamindine]; Sodium Deoxycholate (SDC) [class: Acid]; Ethidium Bromide [class: Phenanthridine]; Methyl Viologen [class: Paraquat]; Tetraphenylphosphonium (TPP) [class: Quaternary Ammonium Compounds (QACs)]; Rhodamine 123 [class: Xanthene]; Synergise; Wex-cide-128. | AdeB/AdeJ family multidrug efflux RND transporter permease subunit |
| pAcsw19-2 | golT | WP_023187989.1 | Copper (Cu); Gold (Au) | MULTISPECIES: copper-translocating P-type ATPase |
| pAHTJR1 | qacEdelta1 | Q7BQY4 | Benzylkonium Chloride (BAC) [class: Quaternary Ammonium Compounds (QACs)]; Ethidium Bromide [class: Phenanthridine]; Acriflavine [class: Acridine]; Chlorhexidine [class: Biguanides]; Pyronin Y [class: Xanthene]; Rhodamine 6G [class: Xanthene]; Methyl Viologen [class: Paraquat]; Tetraphenylphosphonium (TPP) [class: Quaternary Ammonium Compounds (QACs)]; 4;6-diamidino-2-phenylindole (DAPI) [class: Diamindine]; Acridine Orange [class: Acridine]; Sodium Dodecyl Sulfate (SDS) [class: Organo-sulfate]; Sodium Deoxycholate (SDC) [class: Acid]; Crystal Violet [class: Triarylmethane]; Cetrimide (CTM) [class: Quaternary Ammonium Compounds (QACs)]; Cetylpyridinium Chloride (CPC) [class: Quaternary Ammonium Compounds (QACs)]; Dequalinium [class: Quaternary Ammonium Compounds (QACs)] | Quaternary ammonium compound-resistance protein (QacEdelta1) (QacEdelta1multidrug exporter) (Disinfectant resistance protein) (QacEdelta) (Disinfectant resistance protein qacEdelta1) |
| pALWED1.1 | merB1 | WP_033917449.1 | Mercury (Hg); Phenylmercury Acetate [class: Organo-mercury] | MULTISPECIES: alkylmercury lyase |
| pALWED1.1 | merG | WP_005028438.1 | Mercury (Hg); Phenylmercury Acetate [class: Organo-mercury] | MULTISPECIES: sel1 repeat family protein |
| pALWED1.1 | merA | APW48814.1 | Mercury (Hg); Phenylmercury Acetate [class: Organo-mercury] | mercury(II) reductase (plasmid) |
| pALWED1.1 | merP | WP_005028432.1 | Mercury (Hg) | MULTISPECIES: mercuric transport protein periplasmic component |
| pALWED1.1 | merT | WP_005028429.1 | Mercury (Hg) | MULTISPECIES: mercuric ion transporter MerT |
| pALWED1.1 | merR | CAC80887.1 | Mercury (Hg) | mer operon regulatory protein (plasmid) |
| pALWED1.1 | merC | WP_035884597.1 | Mercury (Hg) | MULTISPECIES: hypothetical protein |
| pALWED1.1 | merA | APW48824.1 | Mercury (Hg); Phenylmercury Acetate [class: Organo-mercury] | mercuric ion reductase merA (plasmid) |
| pALWED1.1 | merB1 | CAC80892.1 | Mercury (Hg); Phenylmercury Acetate [class: Organo-mercury] | alkylmercury lyase (organomercurial lyase) (plasmid) |
| pALWED1.1 | merD | WP_033917457.1 | Mercury (Hg) | MULTISPECIES: mercuric resistance transcriptional repressor protein MerD |
| pALWED1.1 | merE | WP_047731872.1 | Mercury (Hg) | MULTISPECIES: mercury resistance protein |
| pALWED1.1 | chrA | WP_089604980.1 | Chromium (Cr) | chromate transporter |
| pALWED1.1 | nrsD/nreB | WP_004723218.1 | Nickel (Ni); Cobalt (Co) | MULTISPECIES: MFS transporter |
| pAP43-OXA58-NDM1 | golT | WP_023187989.1 | Copper (Cu); Gold (Au) | MULTISPECIES: copper-translocating P-type ATPase |
| pC54_001 | qacEdelta1 | Q7BQY4 | Benzylkonium Chloride (BAC) [class: Quaternary Ammonium Compounds (QACs)]; Ethidium Bromide [class: Phenanthridine]; Acriflavine [class: Acridine]; Chlorhexidine [class: Biguanides]; Pyronin Y [class: Xanthene]; Rhodamine 6G [class: Xanthene]; Methyl Viologen [class: Paraquat]; Tetraphenylphosphonium (TPP) [class: Quaternary Ammonium Compounds (QACs)]; 4;6-diamidino-2-phenylindole (DAPI) [class: Diamindine]; Acridine Orange [class: Acridine]; Sodium Dodecyl Sulfate (SDS) [class: Organo-sulfate]; Sodium Deoxycholate (SDC) [class: Acid]; Crystal Violet [class: Triarylmethane]; Cetrimide (CTM) [class: Quaternary Ammonium Compounds (QACs)]; Cetylpyridinium Chloride (CPC) [class: Quaternary Ammonium Compounds (QACs)]; Dequalinium [class: Quaternary Ammonium Compounds (QACs)] | Quaternary ammonium compound-resistance protein (QacEdelta1) (QacEdelta1multidrug exporter) (Disinfectant resistance protein) (QacEdelta) (Disinfectant resistance protein qacEdelta1) |
| pE47_001 | adeB | WP_068930996.1 | Pyronin Y [class: Xanthene]; Sodium Dodecyl Sulfate (SDS) [class: Organo-sulfate]; Acriflavine [class: Acridine]; Benzylkonium Chloride (BAC) [class: Quaternary Ammonium Compounds (QACs)]; Chlorhexidine [class: Biguanides]; 4;6-diamidino-2-phenylindole (DAPI) [class: Diamindine]; Sodium Deoxycholate (SDC) [class: Acid]; Ethidium Bromide [class: Phenanthridine]; Methyl Viologen [class: Paraquat]; Tetraphenylphosphonium (TPP) [class: Quaternary Ammonium Compounds (QACs)]; Rhodamine 123 [class: Xanthene]; Synergise; Wex-cide-128. | AdeB/AdeJ family multidrug efflux RND transporter permease subunit |
| pE47_001 | adeK | WP_033917515.1 | Acridine Orange [class: Acridine]; Safranin O [class: Azin]; Pyronin Y [class: Xanthene]; Sodium Dodecyl Sulfate (SDS) [class: Organo-sulfate] | MULTISPECIES: adeC/adeK/oprM family multidrug efflux complex outer membrane factor |
| pE47_001 | qacEdelta1 | Q7BQY4 | Benzylkonium Chloride (BAC) [class: Quaternary Ammonium Compounds (QACs)]; Ethidium Bromide [class: Phenanthridine]; Acriflavine [class: Acridine]; Chlorhexidine [class: Biguanides]; Pyronin Y [class: Xanthene]; Rhodamine 6G [class: Xanthene]; Methyl Viologen [class: Paraquat]; Tetraphenylphosphonium (TPP) [class: Quaternary Ammonium Compounds (QACs)]; 4;6-diamidino-2-phenylindole (DAPI) [class: Diamindine]; Acridine Orange [class: Acridine]; Sodium Dodecyl Sulfate (SDS) [class: Organo-sulfate]; Sodium Deoxycholate (SDC) [class: Acid]; Crystal Violet [class: Triarylmethane]; Cetrimide (CTM) [class: Quaternary Ammonium Compounds (QACs)]; Cetylpyridinium Chloride (CPC) [class: Quaternary Ammonium Compounds (QACs)]; Dequalinium [class: Quaternary Ammonium Compounds (QACs)] | Quaternary ammonium compound-resistance protein (QacEdelta1) (QacEdelta1multidrug exporter) (Disinfectant resistance protein) (QacEdelta) (Disinfectant resistance protein qacEdelta1) |
| pE47_001 | qacEdelta1 | Q7BQY4 | Benzylkonium Chloride (BAC) [class: Quaternary Ammonium Compounds (QACs)]; Ethidium Bromide [class: Phenanthridine]; Acriflavine [class: Acridine]; Chlorhexidine [class: Biguanides]; Pyronin Y [class: Xanthene]; Rhodamine 6G [class: Xanthene]; Methyl Viologen [class: Paraquat]; Tetraphenylphosphonium (TPP) [class: Quaternary Ammonium Compounds (QACs)]; 4;6-diamidino-2-phenylindole (DAPI) [class: Diamindine]; Acridine Orange [class: Acridine]; Sodium Dodecyl Sulfate (SDS) [class: Organo-sulfate]; Sodium Deoxycholate (SDC) [class: Acid]; Crystal Violet [class: Triarylmethane]; Cetrimide (CTM) [class: Quaternary Ammonium Compounds (QACs)]; Cetylpyridinium Chloride (CPC) [class: Quaternary Ammonium Compounds (QACs)]; Dequalinium [class: Quaternary Ammonium Compounds (QACs)] | Quaternary ammonium compound-resistance protein (QacEdelta1) (QacEdelta1multidrug exporter) (Disinfectant resistance protein) (QacEdelta) (Disinfectant resistance protein qacEdelta1) |
| pOXA58_010030 | qacEdelta1 | Q7BQY4 | Benzylkonium Chloride (BAC) [class: Quaternary Ammonium Compounds (QACs)]; Ethidium Bromide [class: Phenanthridine]; Acriflavine [class: Acridine]; Chlorhexidine [class: Biguanides]; Pyronin Y [class: Xanthene]; Rhodamine 6G [class: Xanthene]; Methyl Viologen [class: Paraquat]; Tetraphenylphosphonium (TPP) [class: Quaternary Ammonium Compounds (QACs)]; 4;6-diamidino-2-phenylindole (DAPI) [class: Diamindine]; Acridine Orange [class: Acridine]; Sodium Dodecyl Sulfate (SDS) [class: Organo-sulfate]; Sodium Deoxycholate (SDC) [class: Acid]; Crystal Violet [class: Triarylmethane]; Cetrimide (CTM) [class: Quaternary Ammonium Compounds (QACs)]; Cetylpyridinium Chloride (CPC) [class: Quaternary Ammonium Compounds (QACs)]; Dequalinium [class: Quaternary Ammonium Compounds (QACs)] | Quaternary ammonium compound-resistance protein (QacEdelta1) (QacEdelta1multidrug exporter) (Disinfectant resistance protein) (QacEdelta) (Disinfectant resistance protein qacEdelta1) |
| pOXA58_010030 | adeB | WP_068930996.1 | Pyronin Y [class: Xanthene]; Sodium Dodecyl Sulfate (SDS) [class: Organo-sulfate]; Acriflavine [class: Acridine]; Benzylkonium Chloride (BAC) [class: Quaternary Ammonium Compounds (QACs)]; Chlorhexidine [class: Biguanides]; 4;6-diamidino-2-phenylindole (DAPI) [class: Diamindine]; Sodium Deoxycholate (SDC) [class: Acid]; Ethidium Bromide [class: Phenanthridine]; Methyl Viologen [class: Paraquat]; Tetraphenylphosphonium (TPP) [class: Quaternary Ammonium Compounds (QACs)]; Rhodamine 123 [class: Xanthene]; Synergise; Wex-cide-128. | AdeB/AdeJ family multidrug efflux RND transporter permease subunit |
| pOXA58_010030 | adeK | WP_033917515.1 | Acridine Orange [class: Acridine]; Safranin O [class: Azin]; Pyronin Y [class: Xanthene]; Sodium Dodecyl Sulfate (SDS) [class: Organo-sulfate] | MULTISPECIES: adeC/adeK/oprM family multidrug efflux complex outer membrane factor |
| pOXA58_010030 | merE | WP_047731872.1 | Mercury (Hg) | MULTISPECIES: mercury resistance protein |
| pOXA58_010030 | merD | WP_033917457.1 | Mercury (Hg) | MULTISPECIES: mercuric resistance transcriptional repressor protein MerD |
| pOXA58_010030 | merB1 | WP_033917456.1 | Mercury (Hg); Phenylmercury Acetate [class: Organo-mercury] | MULTISPECIES: alkylmercury lyase |
| pOXA58_010030 | merA | WP_033917455.1 | Mercury (Hg); Phenylmercury Acetate [class: Organo-mercury] | MULTISPECIES: mercury(II) reductase |
| pOXA58_010030 | merC | WP_035884597.1 | Mercury (Hg) | MULTISPECIES: hypothetical protein |
| pOXA58_010030 | merT | WP_065995582.1 | Mercury (Hg) | mercuric ion transporter MerT |
| pOXA58_010030 | merR | WP_001166626.1 | Mercury (Hg) | MULTISPECIES: Hg(II)-responsive transcriptional regulator |
| pOXA58_010030 | merT | WP_005028429.1 | Mercury (Hg) | MULTISPECIES: mercuric ion transporter MerT |
| pOXA58_010030 | merP | WP_005028432.1 | Mercury (Hg) | MULTISPECIES: mercuric transport protein periplasmic component |
| pOXA58_010030 | merA | WP_033917451.1 | Mercury (Hg); Phenylmercury Acetate [class: Organo-mercury] | MULTISPECIES: mercury(II) reductase |
| pOXA58_010030 | merG | WP_005028438.1 | Mercury (Hg); Phenylmercury Acetate [class: Organo-mercury] | MULTISPECIES: sel1 repeat family protein |
| pOXA58_010030 | merB1 | WP_033917449.1 | Mercury (Hg); Phenylmercury Acetate [class: Organo-mercury] | MULTISPECIES: alkylmercury lyase |
| pOXA58_010055 | golT | WP_023187989.1 | Copper (Cu); Gold (Au) | MULTISPECIES: copper-translocating P-type ATPase |
| pOXA58_010055 | qacEdelta1 | Q7BQY4 | Benzylkonium Chloride (BAC) [class: Quaternary Ammonium Compounds (QACs)]; Ethidium Bromide [class: Phenanthridine]; Acriflavine [class: Acridine]; Chlorhexidine [class: Biguanides]; Pyronin Y [class: Xanthene]; Rhodamine 6G [class: Xanthene]; Methyl Viologen [class: Paraquat]; Tetraphenylphosphonium (TPP) [class: Quaternary Ammonium Compounds (QACs)]; 4;6-diamidino-2-phenylindole (DAPI) [class: Diamindine]; Acridine Orange [class: Acridine]; Sodium Dodecyl Sulfate (SDS) [class: Organo-sulfate]; Sodium Deoxycholate (SDC) [class: Acid]; Crystal Violet [class: Triarylmethane]; Cetrimide (CTM) [class: Quaternary Ammonium Compounds (QACs)]; Cetylpyridinium Chloride (CPC) [class: Quaternary Ammonium Compounds (QACs)]; Dequalinium [class: Quaternary Ammonium Compounds (QACs)] | Quaternary ammonium compound-resistance protein (QacEdelta1) (QacEdelta1multidrug exporter) (Disinfectant resistance protein) (QacEdelta) (Disinfectant resistance protein qacEdelta1) |
| pOXA58_010055 | merR | WP_001166626.1 | Mercury (Hg) | MULTISPECIES: Hg(II)-responsive transcriptional regulator |
| pOXA58_010055 | merT | WP_001294659.1 | Mercury (Hg) | MULTISPECIES: mercuric transporter |
| pOXA58_010055 | merC | WP_035884597.1 | Mercury (Hg) | MULTISPECIES: hypothetical protein |
| pOXA58_010055 | merA | WP_033917455.1 | Mercury (Hg); Phenylmercury Acetate [class: Organo-mercury] | MULTISPECIES: mercury(II) reductase |
| pOXA58_010055 | merB1 | WP_033917456.1 | Mercury (Hg); Phenylmercury Acetate [class: Organo-mercury] | MULTISPECIES: alkylmercury lyase |
| pOXA58_010055 | merD | WP_033917457.1 | Mercury (Hg) | MULTISPECIES: mercuric resistance transcriptional repressor protein MerD |
| pOXA58_010055 | merE | WP_047731872.1 | Mercury (Hg) | MULTISPECIES: mercury resistance protein |
| pOXA58_010055 | adeK | WP_033917515.1 | Acridine Orange [class: Acridine]; Safranin O [class: Azin]; Pyronin Y [class: Xanthene]; Sodium Dodecyl Sulfate (SDS) [class: Organo-sulfate] | MULTISPECIES: adeC/adeK/oprM family multidrug efflux complex outer membrane factor |
| pOXA58_010055 | adeB | WP_068930996.1 | Pyronin Y [class: Xanthene]; Sodium Dodecyl Sulfate (SDS) [class: Organo-sulfate]; Acriflavine [class: Acridine]; Benzylkonium Chloride (BAC) [class: Quaternary Ammonium Compounds (QACs)]; Chlorhexidine [class: Biguanides]; 4;6-diamidino-2-phenylindole (DAPI) [class: Diamindine]; Sodium Deoxycholate (SDC) [class: Acid]; Ethidium Bromide [class: Phenanthridine]; Methyl Viologen [class: Paraquat]; Tetraphenylphosphonium (TPP) [class: Quaternary Ammonium Compounds (QACs)]; Rhodamine 123 [class: Xanthene]; Synergise; Wex-cide-128. | AdeB/AdeJ family multidrug efflux RND transporter permease subunit |
| pR4WN_12CE1 | qacEdelta1 | Q7BQY4 | Benzylkonium Chloride (BAC) [class: Quaternary Ammonium Compounds (QACs)]; Ethidium Bromide [class: Phenanthridine]; Acriflavine [class: Acridine]; Chlorhexidine [class: Biguanides]; Pyronin Y [class: Xanthene]; Rhodamine 6G [class: Xanthene]; Methyl Viologen [class: Paraquat]; Tetraphenylphosphonium (TPP) [class: Quaternary Ammonium Compounds (QACs)]; 4;6-diamidino-2-phenylindole (DAPI) [class: Diamindine]; Acridine Orange [class: Acridine]; Sodium Dodecyl Sulfate (SDS) [class: Organo-sulfate]; Sodium Deoxycholate (SDC) [class: Acid]; Crystal Violet [class: Triarylmethane]; Cetrimide (CTM) [class: Quaternary Ammonium Compounds (QACs)]; Cetylpyridinium Chloride (CPC) [class: Quaternary Ammonium Compounds (QACs)]; Dequalinium [class: Quaternary Ammonium Compounds (QACs)] | Quaternary ammonium compound-resistance protein (QacEdelta1) (QacEdelta1multidrug exporter) (Disinfectant resistance protein) (QacEdelta) (Disinfectant resistance protein qacEdelta1) |
| pR4WN_12CE1 | golT | WP_000536249.1 | Copper (Cu); Gold (Au) | MULTISPECIES: copper-translocating P-type ATPase |
| pR4WN_1BD1 | qacEdelta1 | Q7BQY4 | Benzylkonium Chloride (BAC) [class: Quaternary Ammonium Compounds (QACs)]; Ethidium Bromide [class: Phenanthridine]; Acriflavine [class: Acridine]; Chlorhexidine [class: Biguanides]; Pyronin Y [class: Xanthene]; Rhodamine 6G [class: Xanthene]; Methyl Viologen [class: Paraquat]; Tetraphenylphosphonium (TPP) [class: Quaternary Ammonium Compounds (QACs)]; 4;6-diamidino-2-phenylindole (DAPI) [class: Diamindine]; Acridine Orange [class: Acridine]; Sodium Dodecyl Sulfate (SDS) [class: Organo-sulfate]; Sodium Deoxycholate (SDC) [class: Acid]; Crystal Violet [class: Triarylmethane]; Cetrimide (CTM) [class: Quaternary Ammonium Compounds (QACs)]; Cetylpyridinium Chloride (CPC) [class: Quaternary Ammonium Compounds (QACs)]; Dequalinium [class: Quaternary Ammonium Compounds (QACs)] | Quaternary ammonium compound-resistance protein (QacEdelta1) (QacEdelta1multidrug exporter) (Disinfectant resistance protein) (QacEdelta) (Disinfectant resistance protein qacEdelta1) |
| pR4WN_E10B | qacEdelta1 | Q7BQY4 | Benzylkonium Chloride (BAC) [class: Quaternary Ammonium Compounds (QACs)]; Ethidium Bromide [class: Phenanthridine]; Acriflavine [class: Acridine]; Chlorhexidine [class: Biguanides]; Pyronin Y [class: Xanthene]; Rhodamine 6G [class: Xanthene]; Methyl Viologen [class: Paraquat]; Tetraphenylphosphonium (TPP) [class: Quaternary Ammonium Compounds (QACs)]; 4;6-diamidino-2-phenylindole (DAPI) [class: Diamindine]; Acridine Orange [class: Acridine]; Sodium Dodecyl Sulfate (SDS) [class: Organo-sulfate]; Sodium Deoxycholate (SDC) [class: Acid]; Crystal Violet [class: Triarylmethane]; Cetrimide (CTM) [class: Quaternary Ammonium Compounds (QACs)]; Cetylpyridinium Chloride (CPC) [class: Quaternary Ammonium Compounds (QACs)]; Dequalinium [class: Quaternary Ammonium Compounds (QACs)] | Quaternary ammonium compound-resistance protein (QacEdelta1) (QacEdelta1multidrug exporter) (Disinfectant resistance protein) (QacEdelta) (Disinfectant resistance protein qacEdelta1) |
| pWM98B | merB1 | WP_005028440.1 | Mercury (Hg); Phenylmercury Acetate [class: Organo-mercury] | MULTISPECIES: alkylmercury lyase |
| pWM98B | merG | WP_005028438.1 | Mercury (Hg); Phenylmercury Acetate [class: Organo-mercury] | MULTISPECIES: sel1 repeat family protein |
| pWM98B | merA | WP_043041557.1 | Mercury (Hg); Phenylmercury Acetate [class: Organo-mercury] | mercury(II) reductase |
| pWM98B | merP | WP_005028432.1 | Mercury (Hg) | MULTISPECIES: mercuric transport protein periplasmic component |
| pWM98B | merT | WP_005028429.1 | Mercury (Hg) | MULTISPECIES: mercuric ion transporter MerT |
| pWM98B | merR | KZZ00146.1 | Mercury (Hg) | MerR family transcriptional regulator |
| pWM98B | merB | WP_065811404.1 | Mercury (Hg); Phenylmercury Acetate [class: Organo-mercury]; Methylmercury Acetate [class: Organo-mercury] | alkylmercury lyase |
| pWM98B | merD | WP_010591789.1 | Mercury (Hg) | MULTISPECIES: mercuric resistance transcriptional repressor protein MerD |
| pWM98B | merE | WP_047731872.1 | Mercury (Hg) | MULTISPECIES: mercury resistance protein |
| pRIVM0002 | qacEdelta1 | Q7BQY4 | Benzylkonium Chloride (BAC) [class: Quaternary Ammonium Compounds (QACs)]; Ethidium Bromide [class: Phenanthridine]; Acriflavine [class: Acridine]; Chlorhexidine [class: Biguanides]; Pyronin Y [class: Xanthene]; Rhodamine 6G [class: Xanthene]; Methyl Viologen [class: Paraquat]; Tetraphenylphosphonium (TPP) [class: Quaternary Ammonium Compounds (QACs)]; 4;6-diamidino-2-phenylindole (DAPI) [class: Diamindine]; Acridine Orange [class: Acridine]; Sodium Dodecyl Sulfate (SDS) [class: Organo-sulfate]; Sodium Deoxycholate (SDC) [class: Acid]; Crystal Violet [class: Triarylmethane]; Cetrimide (CTM) [class: Quaternary Ammonium Compounds (QACs)]; Cetylpyridinium Chloride (CPC) [class: Quaternary Ammonium Compounds (QACs)]; Dequalinium [class: Quaternary Ammonium Compounds (QACs)] | Quaternary ammonium compound-resistance protein (QacEdelta1) (QacEdelta1multidrug exporter) (Disinfectant resistance protein) (QacEdelta) (Disinfectant resistance protein qacEdelta1) |
| pRIVM0002 | qacEdelta1 | Q7BQY4 | Benzylkonium Chloride (BAC) [class: Quaternary Ammonium Compounds (QACs)]; Ethidium Bromide [class: Phenanthridine]; Acriflavine [class: Acridine]; Chlorhexidine [class: Biguanides]; Pyronin Y [class: Xanthene]; Rhodamine 6G [class: Xanthene]; Methyl Viologen [class: Paraquat]; Tetraphenylphosphonium (TPP) [class: Quaternary Ammonium Compounds (QACs)]; 4;6-diamidino-2-phenylindole (DAPI) [class: Diamindine]; Acridine Orange [class: Acridine]; Sodium Dodecyl Sulfate (SDS) [class: Organo-sulfate]; Sodium Deoxycholate (SDC) [class: Acid]; Crystal Violet [class: Triarylmethane]; Cetrimide (CTM) [class: Quaternary Ammonium Compounds (QACs)]; Cetylpyridinium Chloride (CPC) [class: Quaternary Ammonium Compounds (QACs)]; Dequalinium [class: Quaternary Ammonium Compounds (QACs)] | Quaternary ammonium compound-resistance protein (QacEdelta1) (QacEdelta1multidrug exporter) (Disinfectant resistance protein) (QacEdelta) (Disinfectant resistance protein qacEdelta1) |
| pRIVM0002 | qacEdelta1 | Q7BQY4 | Benzylkonium Chloride (BAC) [class: Quaternary Ammonium Compounds (QACs)]; Ethidium Bromide [class: Phenanthridine]; Acriflavine [class: Acridine]; Chlorhexidine [class: Biguanides]; Pyronin Y [class: Xanthene]; Rhodamine 6G [class: Xanthene]; Methyl Viologen [class: Paraquat]; Tetraphenylphosphonium (TPP) [class: Quaternary Ammonium Compounds (QACs)]; 4;6-diamidino-2-phenylindole (DAPI) [class: Diamindine]; Acridine Orange [class: Acridine]; Sodium Dodecyl Sulfate (SDS) [class: Organo-sulfate]; Sodium Deoxycholate (SDC) [class: Acid]; Crystal Violet [class: Triarylmethane]; Cetrimide (CTM) [class: Quaternary Ammonium Compounds (QACs)]; Cetylpyridinium Chloride (CPC) [class: Quaternary Ammonium Compounds (QACs)]; Dequalinium [class: Quaternary Ammonium Compounds (QACs)] | Quaternary ammonium compound-resistance protein (QacEdelta1) (QacEdelta1multidrug exporter) (Disinfectant resistance protein) (QacEdelta) (Disinfectant resistance protein qacEdelta1) |
| pRIVM0002 | adeK | WP_033917515.1 | Acridine Orange [class: Acridine]; Safranin O [class: Azin]; Pyronin Y [class: Xanthene]; Sodium Dodecyl Sulfate (SDS) [class: Organo-sulfate] | MULTISPECIES: adeC/adeK/oprM family multidrug efflux complex outer membrane factor |
| pRIVM0002 | adeB | WP_068930996.1 | Pyronin Y [class: Xanthene]; Sodium Dodecyl Sulfate (SDS) [class: Organo-sulfate]; Acriflavine [class: Acridine]; Benzylkonium Chloride (BAC) [class: Quaternary Ammonium Compounds (QACs)]; Chlorhexidine [class: Biguanides]; 4;6-diamidino-2-phenylindole (DAPI) [class: Diamindine]; Sodium Deoxycholate (SDC) [class: Acid]; Ethidium Bromide [class: Phenanthridine]; Methyl Viologen [class: Paraquat]; Tetraphenylphosphonium (TPP) [class: Quaternary Ammonium Compounds (QACs)]; Rhodamine 123 [class: Xanthene]; Synergise; Wex-cide-128. | AdeB/AdeJ family multidrug efflux RND transporter permease subunit |
| pRIVM0051 | qacEdelta1 | Q7BQY4 | Benzylkonium Chloride (BAC) [class: Quaternary Ammonium Compounds (QACs)]; Ethidium Bromide [class: Phenanthridine]; Acriflavine [class: Acridine]; Chlorhexidine [class: Biguanides]; Pyronin Y [class: Xanthene]; Rhodamine 6G [class: Xanthene]; Methyl Viologen [class: Paraquat]; Tetraphenylphosphonium (TPP) [class: Quaternary Ammonium Compounds (QACs)]; 4;6-diamidino-2-phenylindole (DAPI) [class: Diamindine]; Acridine Orange [class: Acridine]; Sodium Dodecyl Sulfate (SDS) [class: Organo-sulfate]; Sodium Deoxycholate (SDC) [class: Acid]; Crystal Violet [class: Triarylmethane]; Cetrimide (CTM) [class: Quaternary Ammonium Compounds (QACs)]; Cetylpyridinium Chloride (CPC) [class: Quaternary Ammonium Compounds (QACs)]; Dequalinium [class: Quaternary Ammonium Compounds (QACs)] | Quaternary ammonium compound-resistance protein (QacEdelta1) (QacEdelta1multidrug exporter) (Disinfectant resistance protein) (QacEdelta) (Disinfectant resistance protein qacEdelta1) |
| pRIVM0051 | adeK | WP_033917515.1 | Acridine Orange [class: Acridine]; Safranin O [class: Azin]; Pyronin Y [class: Xanthene]; Sodium Dodecyl Sulfate (SDS) [class: Organo-sulfate] | MULTISPECIES: adeC/adeK/oprM family multidrug efflux complex outer membrane factor |
| pRIVM0051 | adeB | WP_068930996.1 | Pyronin Y [class: Xanthene]; Sodium Dodecyl Sulfate (SDS) [class: Organo-sulfate]; Acriflavine [class: Acridine]; Benzylkonium Chloride (BAC) [class: Quaternary Ammonium Compounds (QACs)]; Chlorhexidine [class: Biguanides]; 4;6-diamidino-2-phenylindole (DAPI) [class: Diamindine]; Sodium Deoxycholate (SDC) [class: Acid]; Ethidium Bromide [class: Phenanthridine]; Methyl Viologen [class: Paraquat]; Tetraphenylphosphonium (TPP) [class: Quaternary Ammonium Compounds (QACs)]; Rhodamine 123 [class: Xanthene]; Synergise; Wex-cide-128. | AdeB/AdeJ family multidrug efflux RND transporter permease subunit |
| pRIVM0061 | qacEdelta1 | Q7BQY4 | Benzylkonium Chloride (BAC) [class: Quaternary Ammonium Compounds (QACs)]; Ethidium Bromide [class: Phenanthridine]; Acriflavine [class: Acridine]; Chlorhexidine [class: Biguanides]; Pyronin Y [class: Xanthene]; Rhodamine 6G [class: Xanthene]; Methyl Viologen [class: Paraquat]; Tetraphenylphosphonium (TPP) [class: Quaternary Ammonium Compounds (QACs)]; 4;6-diamidino-2-phenylindole (DAPI) [class: Diamindine]; Acridine Orange [class: Acridine]; Sodium Dodecyl Sulfate (SDS) [class: Organo-sulfate]; Sodium Deoxycholate (SDC) [class: Acid]; Crystal Violet [class: Triarylmethane]; Cetrimide (CTM) [class: Quaternary Ammonium Compounds (QACs)]; Cetylpyridinium Chloride (CPC) [class: Quaternary Ammonium Compounds (QACs)]; Dequalinium [class: Quaternary Ammonium Compounds (QACs)] | Experimental validation |
| pRIVM0061 | qacEdelta1 | Q7BQY4 | Benzylkonium Chloride (BAC) [class: Quaternary Ammonium Compounds (QACs)]; Ethidium Bromide [class: Phenanthridine]; Acriflavine [class: Acridine]; Chlorhexidine [class: Biguanides]; Pyronin Y [class: Xanthene]; Rhodamine 6G [class: Xanthene]; Methyl Viologen [class: Paraquat]; Tetraphenylphosphonium (TPP) [class: Quaternary Ammonium Compounds (QACs)]; 4;6-diamidino-2-phenylindole (DAPI) [class: Diamindine]; Acridine Orange [class: Acridine]; Sodium Dodecyl Sulfate (SDS) [class: Organo-sulfate]; Sodium Deoxycholate (SDC) [class: Acid]; Crystal Violet [class: Triarylmethane]; Cetrimide (CTM) [class: Quaternary Ammonium Compounds (QACs)]; Cetylpyridinium Chloride (CPC) [class: Quaternary Ammonium Compounds (QACs)]; Dequalinium [class: Quaternary Ammonium Compounds (QACs)] | Experimental validation |
| pRIVM0061 | qacF | ACS73595.1 | Cetrimide (CTM) [class: Quaternary Ammonium Compounds (QACs)] | QacF |
| pRIVM0061 | adeK | WP_033917515.1 | Acridine Orange [class: Acridine]; Safranin O [class: Azin]; Pyronin Y [class: Xanthene]; Sodium Dodecyl Sulfate (SDS) [class: Organo-sulfate] | MULTISPECIES: adeC/adeK/oprM family multidrug efflux complex outer membrane factor |
| pRIVM0061 | adeB | WP_068930996.1 | Pyronin Y [class: Xanthene]; Sodium Dodecyl Sulfate (SDS) [class: Organo-sulfate]; Acriflavine [class: Acridine]; Benzylkonium Chloride (BAC) [class: Quaternary Ammonium Compounds (QACs)]; Chlorhexidine [class: Biguanides]; 4;6-diamidino-2-phenylindole (DAPI) [class: Diamindine]; Sodium Deoxycholate (SDC) [class: Acid]; Ethidium Bromide [class: Phenanthridine]; Methyl Viologen [class: Paraquat]; Tetraphenylphosphonium (TPP) [class: Quaternary Ammonium Compounds (QACs)]; Rhodamine 123 [class: Xanthene]; Synergise; Wex-cide-128. | AdeB/AdeJ family multidrug efflux RND transporter permease subunit |
| pXBB1-9 | qacEdelta1 | Q7BQY4 | Benzylkonium Chloride (BAC) [class: Quaternary Ammonium Compounds (QACs)]; Ethidium Bromide [class: Phenanthridine]; Acriflavine [class: Acridine]; Chlorhexidine [class: Biguanides]; Pyronin Y [class: Xanthene]; Rhodamine 6G [class: Xanthene]; Methyl Viologen [class: Paraquat]; Tetraphenylphosphonium (TPP) [class: Quaternary Ammonium Compounds (QACs)]; 4;6-diamidino-2-phenylindole (DAPI) [class: Diamindine]; Acridine Orange [class: Acridine]; Sodium Dodecyl Sulfate (SDS) [class: Organo-sulfate]; Sodium Deoxycholate (SDC) [class: Acid]; Crystal Violet [class: Triarylmethane]; Cetrimide (CTM) [class: Quaternary Ammonium Compounds (QACs)]; Cetylpyridinium Chloride (CPC) [class: Quaternary Ammonium Compounds (QACs)]; Dequalinium [class: Quaternary Ammonium Compounds (QACs)] | Quaternary ammonium compound-resistance protein (QacEdelta1) (QacEdelta1multidrug exporter) (Disinfectant resistance protein) (QacEdelta) (Disinfectant resistance protein qacEdelta1) |
| pXBB1-9 | merB1 | WP_033917449.1 | Mercury (Hg); Phenylmercury Acetate [class: Organo-mercury] | MULTISPECIES: alkylmercury lyase |
| pXBB1-9 | merG | WP_005028438.1 | Mercury (Hg); Phenylmercury Acetate [class: Organo-mercury] | MULTISPECIES: sel1 repeat family protein |
| pXBB1-9 | merA | WP_033917451.1 | Mercury (Hg); Phenylmercury Acetate [class: Organo-mercury] | MULTISPECIES: mercury(II) reductase |
| pXBB1-9 | merP | WP_005028432.1 | Mercury (Hg) | MULTISPECIES: mercuric transport protein periplasmic component |
| pXBB1-9 | merT | WP_005028429.1 | Mercury (Hg) | MULTISPECIES: mercuric ion transporter MerT |
| pXBB1-9 | merR | WP_001166626.1 | Mercury (Hg) | MULTISPECIES: Hg(II)-responsive transcriptional regulator |
| pXBB1-9 | merT | WP_001294659.1 | Mercury (Hg) | MULTISPECIES: mercuric transporter |
| pXBB1-9 | merP | WP_006581731.1 | Mercury (Hg) | mercury transporter |
| pXBB1-9 | merC | WP_035884597.1 | Mercury (Hg) | MULTISPECIES: hypothetical protein |
| pXBB1-9 | merA | WP_058952602.1 | Mercury (Hg); Phenylmercury Acetate [class: Organo-mercury] | mercury(II) reductase |
| pXBB1-9 | merB1 | WP_033917456.1 | Mercury (Hg); Phenylmercury Acetate [class: Organo-mercury] | MULTISPECIES: alkylmercury lyase |
| pXBB1-9 | merD | WP_033917457.1 | Mercury (Hg) | MULTISPECIES: mercuric resistance transcriptional repressor protein MerD |
| pXBB1-9 | merE | WP_047731872.1 | Mercury (Hg) | MULTISPECIES: mercury resistance protein |
| pXBB1-9 | adeK | WP_033917515.1 | Acridine Orange [class: Acridine]; Safranin O [class: Azin]; Pyronin Y [class: Xanthene]; Sodium Dodecyl Sulfate (SDS) [class: Organo-sulfate] | MULTISPECIES: adeC/adeK/oprM family multidrug efflux complex outer membrane factor |
| pXBB1-9 | adeB | WP_068930996.1 | Pyronin Y [class: Xanthene]; Sodium Dodecyl Sulfate (SDS) [class: Organo-sulfate]; Acriflavine [class: Acridine]; Benzylkonium Chloride (BAC) [class: Quaternary Ammonium Compounds (QACs)]; Chlorhexidine [class: Biguanides]; 4;6-diamidino-2-phenylindole (DAPI) [class: Diamindine]; Sodium Deoxycholate (SDC) [class: Acid]; Ethidium Bromide [class: Phenanthridine]; Methyl Viologen [class: Paraquat]; Tetraphenylphosphonium (TPP) [class: Quaternary Ammonium Compounds (QACs)]; Rhodamine 123 [class: Xanthene]; Synergise; Wex-cide-128. | AdeB/AdeJ family multidrug efflux RND transporter permease subunit |
